# High-CO_2_ Levels Rather than Acidification Restrict *Emiliania huxleyi* Growth and Performance

**DOI:** 10.1007/s00248-022-02035-3

**Published:** 2022-05-27

**Authors:** Víctor Vázquez, Pablo León, Francisco J. L. Gordillo, Carlos Jiménez, Iñiguez Concepción, Kevin Mackenzie, Eileen Bresnan, María Segovia

**Affiliations:** 1 Department of Ecology, Faculty of Sciences, University of Málaga, Boulevard Louis Pasteur S/N, 29071 Málaga, Spain; 2 Marine Laboratory, Marine Scotland Science, 375 Victoria Road, Aberdeen, AB11 9DB UK; 3 Institute of Medical Sciences, University of Aberdeen, Foresterhill, Aberdeen, AB25 2ZD UK

**Keywords:** *Emiliania huxleyi*, Ocean acidification, Calcification, pCO_2_, Photochemistry, Phytoplankton, Stress, Coccolithophores

## Abstract

The coccolithophore *Emiliania huxleyi* shows a variety of responses to ocean acidification (OA) and to high-CO_2_ concentrations, but there is still controversy on differentiating between these two factors when using different strains and culture methods. A heavily calcified type A strain isolated from the Norwegian Sea was selected and batch cultured in order to understand whether acclimation to OA was mediated mainly by CO_2_ or H^+^, and how it impacted cell growth performance, calcification, and physiological stress management. *Emiliania huxleyi* responded differently to each acidification method. CO_2_-enriched aeration (1200 µatm, pH 7.62) induced a negative effect on the cells when compared to acidification caused by decreasing pH alone (pH 7.60). The growth rates of the coccolithophore were more negatively affected by high pCO_2_ than by low pH without CO_2_ enrichment with respect to the control (400 µatm, pH 8.1). High CO_2_ also affected cell viability and promoted the accumulation of reactive oxygen species (ROS), which was not observed under low pH. This suggests a possible metabolic imbalance induced by high CO_2_ alone. In contrast, the affinity for carbon uptake was negatively affected by both low pH and high CO_2_. Photochemistry was only marginally affected by either acidification method when analysed by PAM fluorometry. The POC and PIC cellular quotas and the PIC:POC ratio shifted along the different phases of the cultures; consequently, calcification did not follow the same pattern observed in cell stress and growth performance. Specifically, acidification by HCl addition caused a higher proportion of severely deformed coccoliths, than CO_2_ enrichment. These results highlight the capacity of CO_2_ rather than acidification itself to generate metabolic stress, not reducing calcification.

**Supplementary Information:**

The online version contains supplementary material available at 10.1007/s00248-022-02035-3.

## Introduction

Over the twenty-first century, the ocean is projected to transition to unprecedented conditions relative to preindustrial levels [1]. The worst case-scenario (Representative Concentration Pathway RCP 8.5) [1, 2] predicts an atmospheric pCO_2_ increase above 1000 µatm with a concomitant reduction of 0.4 units in seawater pH in the upper layer of the ocean with respect to preindustrial levels; this process is known as ocean acidification (OA). OA is already altering the carbonate system (comprising the proportions of the different inorganic carbon forms maintained by the carbonate pump, the carbonate counter pump, and the solubility pump in the ocean) that controls seawater pH [3]. Carbon speciation is predicted to be highly affected, with an expected 17% increase in bicarbonate ions (HCO_3_^−^) and a 54% decrease in carbonate (CO_3_^2−^) concentrations in equilibrium [4]. Thereupon, the saturation state of calcite (Ω_calcite_) and the rain ratio (the ratio of calcite precipitation to organic matter production, RR) will also change [5, 6].

Increased CO_2_ concentration in seawater, and its accompanying acidification, may benefit some phytoplanktonic species while being detrimental to others. Calcifying organisms such as coccolithophores are suggested to be strongly impacted by OA [7]. Coccolithophores take up inorganic carbon (C_i_) and produce both particulate organic carbon (POC) through photosynthesis and particulate inorganic carbon (PIC, CaCO_3_) via calcification [5]. The coccolithophore *Emiliania huxleyi* is a major calcifier in the world’s oceans and can account for 20% to total organic carbon fixation [5, 8] and up to 50% of CaCO_3_ flux to marine sediments [9, 10]. Its abundance and calcifying activity result in the paramount importance of this phytoplankton species [11, 12]. It contributes to the regulation of CO_2_ exchange across the ocean–atmosphere interface and to the biogeochemical cycles through the RR and the production of CO_2_ during calcification by means of the carbonate counter pump [5]. It also depends on the Ω_calcite_ since it is relevant to the maintenance of coccoliths after exocytosis [13]. *E. huxleyi* has a pangenome that provides this species with a high genome variability [14]. This is reflected in a plethora of alternative metabolic traits, a consequence of the ample metabolic repertoire displayed [14, 15]. Such genome variability underpins its capacity to thrive in habitats ranging from the equator to the subarctic, forming extensive blooms under a large variety of environmental conditions that can be seen from outer space [14, 16].

The response of *E. huxleyi* to carbonate chemistry variations has been studied both in the field using natural populations and in the laboratory using monocultures [7, 17–21]. When exposed to elevated CO_2_ and concomitantly low pH, *E. huxleyi* reduced its growth rate and its level of calcification in most of the experiments, leading to thinner coccospheres [22] and thus, imposing high stress to the cells that compromises their viability [15, 23]. However, there is still controversy on whether OA will promote or reduce calcification rates, since some experiments with this same species have resulted in conflicting responses [19, 22, 24–26]. For instance, it has been recently reported that some species of coccolithophores are able to maintain relatively constant ratios of calcification-to-photosynthesis under conditions of high CO_2_ by maintaining pH homeostasis at the site of coccolith forming vesicles [27].

Cell production has been described to be either unaffected or stimulated by increased pCO_2_ [20, and references therein]; however, calcification typically decreases with malformations of coccoliths being commonly observed [13, 28, 29]. Bach et al. [30] suggested that biomass production was stimulated by increased CO_2_ at sub-saturating conditions for photosynthesis, whereas calcification was specifically responsive to the associated decrease in pH. The “CO_2_ or pH issue” has been discussed for long regarding phytoplankton responses to OA, especially in coccolithophores [29]. Such differential CO_2_ and/or pH effects on biomass production and calcification seem to be due to acidification distorting ion homeostasis, thus shifting the metabolism from oxidative to reductive pathways [31, 32] and from HCO_3_^−^ use to CO_2_ diffusive-only C uptake [33]. Differences also largely depend on the specificity of the strain, its calcification ability, and its life cycle [33].

The aim of the present work was to investigate the response of *E. huxleyi* (RCC 1226) from the Norwegian Sea to acidification, either by using increased pCO_2_ condition or by acidification of the media by adding HCl. Our target was to differentiate between CO_2_ or H^+^ ions effects on cell growth performance, calcification, and physiological stress management. For this purpose, we analysed the effects of the two acidification methods on: (1) cell abundance, (2) cell viability, (3) the photosynthetic and C acquisition performance, (4) oxidative stress, (5) calcification, and (6) morphology of the coccospheres.

## Methods

### Culture Conditions and Experimental Set-Up

*Emiliania huxleyi* (Lohmann) Hay *et* Mohler (RCC 1226) cells were provided by the Roscoff Culture Collection, France. This is a heavily calcified type A strain isolated from the Atlantic Ocean close to the Norwegian coast in July 1998. Cells were grown in 3 L sterile Perspex cylinders (Plexiglas XT® 29,080) in artificial seawater medium [34] enriched with f/2 nutrients [35]. Cultures were grown at 16 °C, 120 µmol photons m^−2^ s^−1^ photosynthetic active radiation (PAR, 400–700 nm), and at a 14:10 h light: darkness photoperiod. Irradiance was provided by fluorescent tubes of daylight type Osram Sylvania standard truelight 18 W, as measured by a submersible micro quantum sensor (US-SQS/L, Walz, Germany) connected to a Li-COR 250A radiometer. Cells were exposed to low pH (7.7, LP) by either aerating with air enriched with CO_2_ to 1200 µatm (HC-LP, high carbon and low pH) or by HCl additions in combination with non-enriched aeration (LC-LP, low carbon and low pH). The control condition consisted of non-acidified, non-enriched cultures at 400 µatm CO_2_ (LC-HP, low carbon and high pH). All treatments, including the control, consisted of triplicate cultures continuously stirred with a magnetic bar and aerated to ensure homogeneity without mechanical stress and to avoid cell shading. HC levels of CO_2_ were obtained by mixing atmospheric air with pure CO_2_ (Biogon®, Linde, Germany) to achieve 1200 µatm pCO_2_ inlet flow in each culture was measured by non-dispersive infrared analysis by using a CO_2_ gas analyser (LI-820, Li-COR) at 200 mL min^−1^. The air was filtered through Millipore 0.2 µm fiberglass filters (Merck, Germany). For lowering the pH with HCl, the required volume of 12 N HCl (Merck, Germany) was added to the culture medium until pH = 7.7 was reached prior to cells inoculation. The medium and stock cultures were pre-acclimated to the different pCO_2_ and pH conditions for 72 h in order to avoid transient effects [36] under the conditions described just above. The experiment lasted 9 days, and the 3 L cultures were sampled by extracting 175 mL on days 2, 4, 7, and at the end of the experiment, thus the remaining volume before last sampling was 82.5% of the initial.

### Carbonate System (pCO_2_, DIC, Ω_calcite_, pH, Alkalinity)

pCO_2_, DIC, and Ω_calcite_ were calculated from daily measurements of pH, temperature, salinity, and total alkalinity (TA) using the CO_2_Calc software [37] fitted using GEOSECS constants. pH was measured in all the cultures by using a pH-meter (CRISON Basic 20 +) calibrated daily using the NBS scale. Salinity was measured with a conductivity meter (CRISON 524). The accuracy of the pH-meter and conductivity meter were ± 0.01 pH units and ± 1.5%, respectively. TA was measured using the Gran’s potentiometric method [38]. No certified reference standards were used.

### Cell Abundance and Growth Rates

Cell density was determined using an Accuri™ C6 flow cytometer (BD Biosciences, USA) equipped with an air-cooled laser providing 15 mW at 488 nm and with a standard filter set-up by using 1 mL samples. The trigger was set on red fluorescence and samples were analysed for 90 s at an average flow rate of 14 μL min^−1^. Cells were discriminated on the basis of the side-scatter signal (SSC) *versus* chlorophyll [39, 40].

The growth rate (*µ*) was calculated by fitting the cell density data to the logistic growth model (Eq. 1):

1 $$Ln\left(\frac{\left(K-N\right)}{N}\right)=Ln\left(\left(K-{N}_{0}\right)-1\right)-\mu t$$

where *K* refers to the loading capacity, *N* is the cell density at any given time, *N*_0_ is the cell density at time 0, *µ* is the intrinsic growth rate, and *t* is the time (in days). The logistic model was preferred over the exponential model because the cultures reached the stationary phase.

### Chlorophyll a (Chl a) Concentration and In Vivo Chl a Fluorescence Associated to PSII

Samples of 5 mL were collected from each culture, centrifuged at 4000 g, and the pellet snap frozen in liquid nitrogen and kept at − 80 °C until analysis. Pellets were incubated overnight at 4 °C in N,N-dimethylformamide (Sigma-Aldrich, USA) for Chl *a* extraction. The concentration was determined spectrophotometrically and calculated accordingly [41].

The optimal quantum yield (*F*_V_/*F*_m_) for charge separation in photosystem II (PSII) is frequently used as an indicator of photoinhibition, reflecting the general status. In vivo chlorophyll *a* fluorescence of PSII was determined by using a pulse amplitude modulated fluorometer Water-PAM (Heinz Waltz, Effeltrich, Germany) as described by Schreiber et al. [42]. *F*_0_ and *F*_m_ were determined in 10-min dark-adapted freshly taken 2 mL culture samples, to ensure oxidation of primary quinone acceptor (Q_A_), to obtain the *F*_V_/*F*_m_. *F*_V_ is the variable fluorescence of dark-adapted algae when all the reaction centres are opened as *F*_m_ − *F*_0_. *F*_m_ is the maximal fluorescence intensity with all PSII reaction centres closed obtained after an intense actinic saturation light pulse > 4000 µmol photons m^−2^ s^−1^, and *F*_0_ is the basal fluorescence (minimal fluorescence) of dark-adapted after 10 min. Using the software WinControl-3.25, rapid light curves (RLCs) were constructed and fitted to the nonlinear least-squares regression model of Eilers and Peeters [43] to obtain the initial slope of the curve related to the photosynthetic light-harvesting efficiency (*α*_ETR_) (as an estimator of photosynthetic efficiency) and the relative maximal electron transport rate (*rETR*_max_). The actinic light intensities were selected according to the saturation pattern and measured in the PAM cuvette using a US-SQS/L micro quantum sensor (Walz) attached to a Licor 250-A radiometer. The light requirement for saturating photosynthetic rate (*E*_k_) and the maximum irradiance before photoinhibition of *rETR* was observed (*E*_opt_) were derived from *rETR*_max_ and *α* and *ß* slopes, respectively, where *ß* is the slope of *rETR* decay at high irradiance.

### Cell Viability and Reactive Oxygen Species (ROS)

Cell stress was studied by using the cellular green fluorescence emission of specific probes (Invitrogen, USA) added to samples cultured at each treatment [44]. Cell viability was assessed with fluorescein diacetate (FDA), and 0.4 µL of a 0.09 µM working stock was added to 1 mL samples. FDA is a nonpolar, non-fluorescent stain, which diffuses freely into cells. Inside the cell, the FDA molecule is cleaved (hydrolysed) by nonspecific esterases to yield the fluorescent product fluorescein and two acetates. Accumulations of fluorescein are the result of intracellular esterase activity and thus indicate metabolic activity and therefore cell viability. ROS were assayed with carboxy-H_2_DFFDA, a cell-permeable fluorescent indicator that is non-fluorescent until oxidation by ROS occurs within the cell. H_2_DFFDA detects intracellular ROS species, and despite its lack of specificity, it has been proven very useful and reliable for assessing the overall oxidative stress being oxidized by any possible radical with oxidative activity [44]. ROS detection was performed after 15 µL of a 2 mM working stock of carboxy-H_2_DFFDA were added to 1 mL samples. Samples were incubated at 16 °C in darkness for 120 min under gentle shaking. Fluorescence was measured using an Accuri™ C6 flow cytometer (BD Biosciences, USA). Counts were triggered using forward scatter (FSC) signals.

### Substrate Dependent Kinetics of Inorganic C Fixation

A ^14^C-based method was used to estimate the substrate dependent kinetics of inorganic C fixation based on Tortell et al. [45]. These measurements were conducted through short-term incubations of 10 min over a range of external C concentrations in buffered seawater (20 mM Bicine, pH 8.0). Prior to the beginning of the experiments, inorganic C was removed from the assay buffer by purging 20 mL aliquots with CO_2_-free air for at least 3 h [46, 47]. 1.5 mL of phytoplankton aliquots in C-free buffer were dispensed into polypropylene microcentrifuge tubes and placed in a custom-made, temperature-controlled glass chamber (16 °C). The incubation tubes were illuminated from the side with 600 µmol photons m^−2^ s^−1^ provided by a fluorescent tube of daylight type Osram Sylvania standard truelight 18 W. To initiate measurements, various amounts of 6 mM H^12^CO_3_^−^ (0.108 g HCO_3_^−^  + 20 mL of CO_2_-free water + 30 µL NaOH 4 N) and H^14^CO_3_^−^ (DHI, Denmark) (specific activity vial: 2.18 × 10^9^ Bq · mmol^−1^; final specific activity: 0.055 × 10^9^ Bq · mmol^−1^; 2 mL stock of HCO_3_^−^ cold + 0.2 mL ampoule of ^14^C (total 2.2 mL)) were added to each tube. The ^14^C/^12^C additions were adjusted to yield a final concentration of total inorganic carbon ranging from ≈ 50 to 4,000 µM, with a final specific activity of 0.055 × 10^9^ Bq mmol^−1^. After 10 min of incubation, 500 µL of each tube were rapidly transferred into 500 µL of 6 N HCl in 20 mL scintillation vials and vortexed. Vials were then placed on a shaker table to degas evolved ^14^CO_2_ for at least 12 h. After this time, ^14^C activity of the samples was measured after adding 10 mL of scintillation cocktail (Ultima Gold, Perkin Elmer, USA) using a liquid scintillation counter (Packard Tri Carb Liquid Scintillation Analyser, Model 1900 A, Perkin Elmer, USA) with automatic quench correction. Background activity levels in cell-free blanks were subtracted from all samples.

Kinetic parameters *V*_max_ and *K*_m_ were derived from the ^14^C data using nonlinear, least-squares regression of the hyperbolic Michaelis–Menten equation (Eq. 2):

2 $$V={V}_{\mathrm{max}}*S/(S+{K}_{\mathrm{m}})$$

where *V* is the rate of C fixation at any given external C concentrations (*S*), and *V*_max_ is the maximal rate of C fixation [note that maximal C fixation rates obtained from this analysis are not directly comparable to steady-state C uptake rates measured in traditional (12–24 h) ^14^C-incubation experiments. The 10-min rates reflect the total cellular capacity for C fixation, while longer-term rates include a significant contribution of respiration and organic C release, and cell death]. *K*_m_ was the concentration of C for half *V*_max_. Carbon fixation rates were normalized by previously obtained cell counts.

### Elemental Composition

Total particulate carbon (TPC) was measured using a C:H:N elemental analyser (Perkin-Elmer 2400 CHN). Twenty-five millilitres of each culture were gently filtered through pre-combusted (4.5 h, 500 °C) GF/F filters (Whatman) and dried at 60 °C for 24 h. For the determination of POC, the protocol was the same as for TPC, except those filters were fumed with saturated HCl overnight before analysis. PIC was assessed as the difference between TPC and POC.

### Stable δ^13^C Isotopic Determination

The value of δ^13^C is used as a proxy of HCO_3_^−^ *versus* CO_2_ only used by an aquatic primary producer, the former requiring a carbon concentrating mechanism (CCM). Typically, a value below (more negative than) − 30‰ indicates an inactive or absent CCM. However, this reference value should be taken cautiously, since it can be influenced by the specific δ^13^C value of ribulose-1,5-bisphosphate carboxylase-oxygenase (RuBisCO) for CO_2_ fixation in a given species. The abundance of ^13^C relative to ^12^C in *E. huxleyi* samples was determined by mass spectrometry using a DELTA V Advantage (Thermo Electron Corporation, USA) Isotope Ratio Mass Spectrometer (IRMS) connected to a Flash EA 1112 CNH analyser. δ^13^C isotopic discrimination in the microalgae samples (δ^13^C_sample_) was expressed in the unit notation as deviations from the ^13^C/^12^C ratio of the Pee-Dee Belemnite CaCO_3_ (PDB, which is the same as VPDB) calculated according to (Eq. 3):

3 $${\updelta }^{13}\mathrm{C}\left(\mathrm{\permil }\right)=[({\updelta }^{13}\mathrm{C}/{\updelta }^{12}\mathrm{C}{)}_{\mathrm{sample}}/({\updelta }^{13}\mathrm{C}/{\updelta }^{12}\mathrm{C}{)}_{\mathrm{PDB}}-1]\bullet {10}^{3}$$

To determine the isotopic composition of dissolved inorganic carbon (δ^13^C_DIC_), 25 mL from each cylinder were filtered (Whatman GF/F). Measurements of δ^13^C_DIC_ were performed with the same IRMS mentioned above connected to a GasBench II (Thermo Electron Corporation) system. The δ^13^C_sample_ was corrected by δ^13^C_DIC_ values from the medium, previously tested in a TOC-L analyser.

### pH Drift

A pH drift experiment was carried out to determine if *E. huxleyi* can use HCO_3_^−^ as a source of inorganic carbon. The ability of algae to raise the pH of the medium to more than 9.0 is considered as evidence of their ability to use HCO_3_^−^. Samples were placed in 100 mL glass bottles filled (without leaving a head space) with 0.2 µL filtered seawater enriched with f/2 nutrients, and tightly sealed to avoid gas exchange. To obtain a complete homogenization of the medium, a magnetic bar was placed into each bottle and continuous stirring was provided by a magnetic stirrer. Samples were exposed to continuous illumination provided by white fluorescent tubes at saturating light. The pH was recorded by introducing a glass electrode through the lid of the glass bottle each 4–5 h until a stable reading was reached. Measurements were carried out until no further increase of the pH was detected.

### Morphometric and Data Analysis of Coccoliths and Coccospheres

Size and morphological features of the cells were analysed using scanning electron microscopy (SEM). Samples of the different treatments (250 µL, 500 µL, and 1000 µL, depending on cell abundance) were filtered using Millipore Isopore™ hydrophilic polycarbonate membranes (RTTO01300) of 13 mm in diameter, and a pore size of 0.8 µm, using a vacuum pump under low pressure (< 200 mbar). Filters were rinsed with buffered distilled water to remove salt and then air dried overnight, mounted on aluminium SEM stubs, sputter coated with gold/palladium, and subsequently examined using a Zeiss EVO MA10 SEM.

The coccoliths were visually classified according to four morphological categories to estimate their degree of malformation [48, 49] (Fig. 6). The first category corresponds to normal coccoliths with all segments connected and forming an oval ring. The next three categories represent stages with increasing malformation signs characterized by a reduced symmetry, an altered shape of some of the elements, and reduced distal shield elements. Specifically, the second category corresponds to slightly malformed coccoliths, with less than 5 T-segments not well connected to others. The third category corresponds to malformed coccoliths where more than 5 T-segments are disconnected or not entirely formed. The fourth corresponds to fragmented coccoliths, in which parts of the coccoliths are missing. Category 4 is considered as severe malformation. The damaged coccoliths were measured only if their “reference points” could be unequivocally determined. Approximately 30 coccospheres and 30 coccoliths of each treatment were analysed (i.e., HC-LP, LC-HP, and LC-LP). The mean values of each parameter were considered constant when there were more than 20 coccospheres and coccoliths measurable per sample [50]. As for the coccoliths, all the morphometric measurements were performed on the distal shield of flat lying *E. huxleyi* placoliths (see Supplementary Fig. S2). Measurements included the length of the distal shield (*DL*), the width of the distal shield (*DW*), the length of the central area (*CAL*), and the width of the central area (*CAW*). *CAL* and *CAW* could not be determined in cases where the coccolith was lying upside-down on the filter. In addition, the number of segments or elements that form the distal shield were recorded. The surface area of the distal shield (*DSA*) was estimated with the values of *DL* and *DW* [51] (Eq. 4):

4 $$DSA=\pi \cdot \frac{DL\cdot DW}{4}$$

The outer shield length (*OSL*) was calculated assuming an elliptical shape of coccolith, such as (Eq. 5):

5 $$OSL=\frac{DL-CAL+DW-CAW}{4}$$

In addition, the calculation of the surface area of central shield (*CSA*) is proposed taking the values of *CAL* and *CAW*, such as (Eq. 6):

6 $$CSA=\pi \cdot \frac{CAL\cdot CAW}{4}$$

The width of the tube (protococcolith ring) (*TW*) varies between coccoliths of *E. huxleyi*, from slightly calcified coccoliths in which the central area is wide and the tube is narrow to very calcified coccoliths in which the central area is almost closed. To obtain a size independent parameter to measure this degree of calcification variation, we used relative tube width (*TW*_relative_) (Eq. 7). This parameter is used here as a calcification index. This ratio is dimensionless and should be size-independent.

7 $${TW}_{\mathrm{relative}}=\frac{2\bullet TW}{DW}$$

Coccoliths mass (*m*) has also been used as an indicator of the impact of OA on coccolithophores [52, 53] as (Eq. 8):

8 $$m=2.7\bullet {k}_{\mathrm{s}}\bullet {DL}^{3}$$

where *k*_s_ is a shape dependant constant, *K*_s_ = 0.07 *TW*_relative_, and *DL* distal shield length.

The roundness of the distal shield (*DR*) (Eq. 9) and the roundness of the central area (*CAR*) (Eq. 10) were calculated, from the ratio of their width and length measurements [54] as:

9 $$DR=DW/DL\bullet 100$$

10 $$CAR=CAW/CAL\bullet 100$$

As for the coccospheres, two measurements were made, one of them corresponding to the greater length *L* and another to the shorter length *W*.

Measurements were taken from SEM micrographs which were processed and analysed using the software Fiji-ImageJ 1.49v software [55, 56] (National Institute of Health, USA).

### Statistical Analyses

Statistical significance of treatments was analysed by performing split-plot ANOVAs (SPANOVAs, or mixed-model ANOVAs) followed by post hoc Sidak or Tukey and Bonferroni tests, respectively (considering *p* < 0.05 as significant). When appropriate, data were specifically tested for significant differences (*p* < 0.05) induced by the treatments by using 1-way ANOVAs and/or Student’s *t*-tests, as well as Pearson’s product-moment correlations. All analyses were performed using the general linear model (GLM) procedure. Data were previously checked for normality (by Shapiro-Wilks’ test), homoscedasticity (by Cochran’s and Levene’s tests), and sphericity (by Mauchly’s and/or Bartlett’s tests). Variables met all criteria mentioned above. Statistical analyses were performed using the software SPSS v.22 (IBM statistics) and R-studio.

## Results

### Carbonate System

The carbonate system data are shown in Table 1. The initial pCO_2_ levels were 1233 ± 59 µatm in the “high-CO_2_” treatment (HC-LP) and 456 ± 39 µatm and 718 ± 77 µatm in the control (LC-HP) and “low-pH” (LC-LP) treatments, respectively. The biological activity promoted a gradual change in the carbonate chemistry conditions from the start to the end of the experiment. Accordingly, pCO_2_ decreased between day 1 (d_1_) and day 4 (d_4_) in all treatments, reaching a minimum level of ~ 300 µatm in the LC-LP and 200 µatm in LC-HP, as a consequence of the gradual decline in total alkalinity derived from calcification and cell division. Values for pCO_2_ did not show significant differences between the two low CO_2_ treatments (LC-HP and LC-LP) for the rest of the experiment (see Supplementary Table S4).

**Table 1:** Corrected δ^13^C isotopic discrimination (δ^13^C_microalgae_) and pH compensation point for *E. huxleyi* under the different experimental conditions on day 9; HC-LP (pCO_2_: 1000–1200 µatm; pH: 7.6–7.8), LC-HP (pCO_2_: 380–390 µatm; pH: 8.2), and LC-LP (pCO_2_: 380–390 µatm; pH: 7.6–7.8). Values are mean ± SD. Significant differences between treatments are indicated by different letters (*p* < 0.05)

Treatment	δ^13^C_microalgae_ (‰)	pH compensation point
HC-LP	− 28.37 ± 1.10^a^	8.02 ± 0.03^a^
LC-HP	− 16.07 ± 0.27^b^	9.70 ± 0.07^b^
LC-LP	− 18.17 ± 1.15^b^	9.17 ± 0.03^c^

**Fig. 1:**
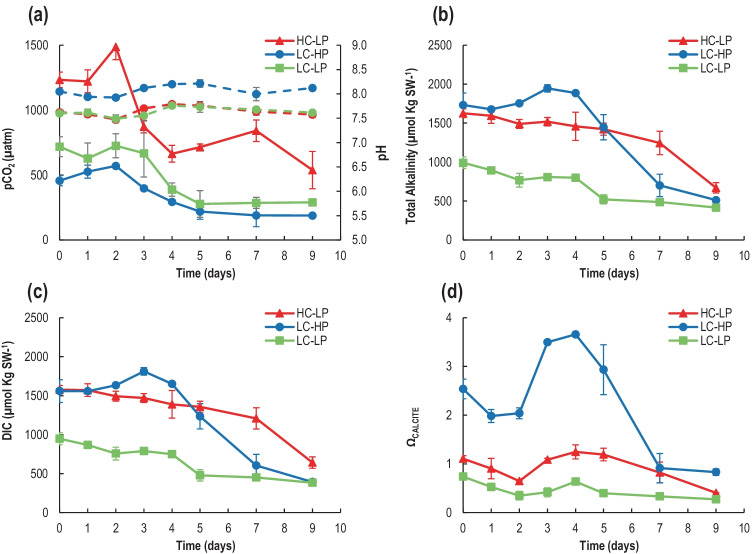
Measured pH (dotted line) (**a**) and total alkalinity (**b**), and calculated pCO_2_ (solid line) (**a**), dissolved inorganic carbon (DIC) (**c**), and calcite saturation estate (Ω_calcite_) (**d**), in the different treatments: HC-LP (pCO_2_: 1000–1200 µatm; pH: 7.6–7.8), LC-HP (pCO_2_: 380–390 µatm; pH: 8.2), and LC-LP (pCO_2_: 380–390 µatm; pH: 7.6–7.8). Values are mean ± SD (*n* = 3). Significant differences between treatments are indicated by different letters for any given time (*p* < 0.05)

Ω_calcite_ was significantly higher in the control than in the two other treatments (*p* < 0.05, Supp. Table S4), reaching a maximum value of 3.66 ± 0.04 at d_4_, in contrast to 1.25 ± 0.14 and 0.63 ± 0.05 in the high-CO_2_ and the low-pH treatments, respectively. In agreement with the two acidification methods, TA and DIC did not present significant differences between the control and the high-CO_2_ treatments at d_0_, but were higher than in the low-pH treatment (Table S4). TA and DIC values gradually decreased between d_0_ and d_9_ in all treatments, to finally drop to the minimum values by the end of the culture period. The concentration of HCO_3_^−^ showed a similar pattern to TA/DIC when acidification was produced by pCO_2_ and not by HCl, being similar at both pH 8.2 and 7.6–7.8 until day 5 (Fig. 1).

### Cell Density and Growth Rates

**Fig. 2:**
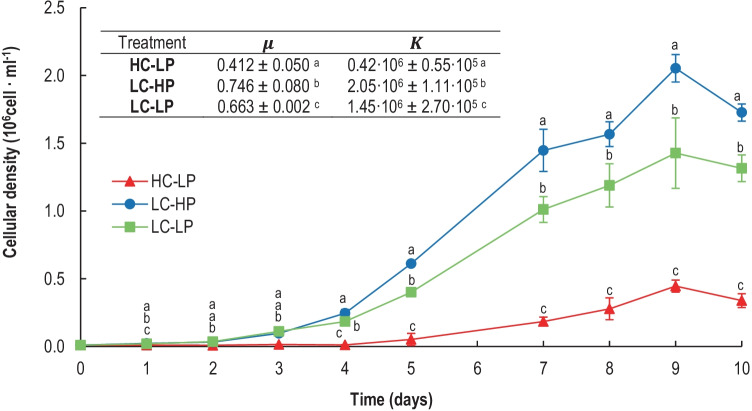
Cellular abundance (10^6^ cell mL^−1^), net growth rates (*µ*, d^−1^), and loading capacity of the culture (*K*, 10^6^ cell mL^−1^) of *E. huxleyi* cultures calculated with the logistic model of growth under HC-LP (1000–1200 µatm and pH 7.6–7.8), LC-HP (380–390 µatm and pH 8.1), and LC-LP (380–390 µatm and pH 7.6–7.8). Values are mean ± SD (*n* = 3). Significant differences between treatments are indicated by different letters for any given time (*p* < 0.05)

All cultures reached the highest densities after d_9_ (Fig. 2). The maximal cell densities of the cultures (*K*) ranged between 0.24 × 10^6^ cells mL^−1^ (in HC-LP, CO_2_-enriched cultures) and 2.05 × 10^6^ cells mL^−1^ (in LC-HP, control conditions). The intrinsic growth rate (*µ*) was lower in HC-LP (0.412 d^−1^) than in LC-HP (0.746 d^−1^) (*p* < 0.05, Table S4). Both *K* and *µ* were mostly affected by high pCO_2_ (HC-LP); *K* was reduced by 80% and *µ* by 45% with respect to control conditions (LC-HP). When pH was decreased by acidification with HCl (LC-LP), the reductions of *K* and *µ* were only 30% and 11%, respectively, with respect to the control.

### Cell Viability and Oxidative Stress

**Fig. 3:**
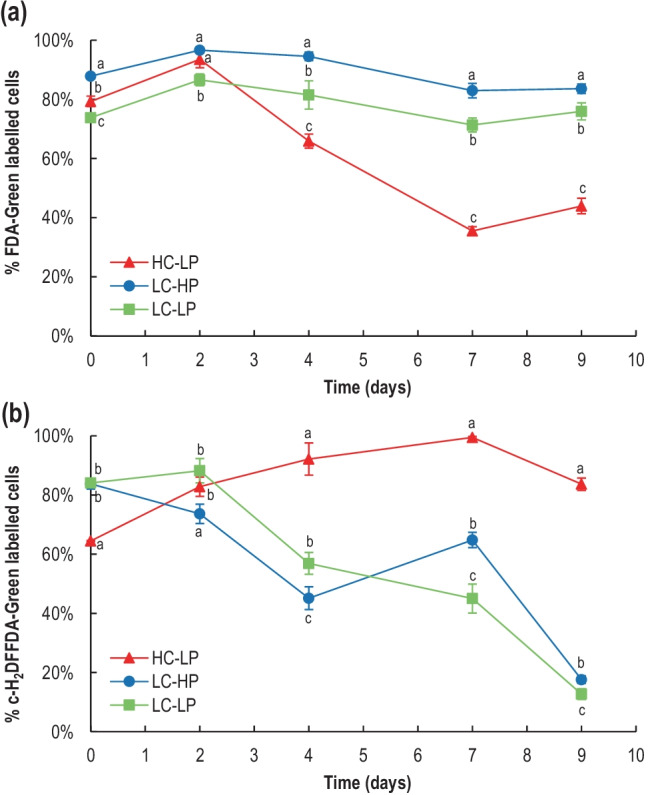
Variation of (**a**) cell viability measured as FDA-green fluorescence labelled *E. huxleyi* cells, and (**b**) reactive oxygen species (ROS) measured as c-H_2_DFFDA-green fluorescence labelled *E. huxleyi* cells in HC-LP (1000–1200 µatm and pH 7.6–7.8), LC-HP (380–390 µatm and pH 8.1), and LC-LP (380–390 µatm and pH 7.6–7.8). Values are mean ± SD (*n* = 3). Significant differences between treatments are indicated by different letters for any given time (*p* < 0.05)

Cell viability showed significant differences between treatments (*p* < 0.05, Table S4; Fig. 3a). In the control conditions (LC-HP), cell viability was ~ 90% along the entire experiment. Acidification with HCl (LC-LP) reduced cell viability by an extra 10% compared to the control, i.e., 80% were still viable. However, in CO_2_-enriched cultures (HC-LP), the cell viability sharply declined from 48 h onwards, and only 35% of the cells remained alive by the end of the experiments. General oxidative stress was high in all treatments at initial times (Fig. 3b), most likely indicating a temporary stress by the dilution effect according to ROS accumulation. Highest ROS content occurred in HC-LP (~ 90% green fluorescence labelled cells) from d_4_ to d_9_. However, in LC-HP and LC-LP, only ~ 10% of the cells showed ROS green fluorescence by d_9_, and thus in LC, only a small proportion of the cells were stressed.

### Chl a Concentration and In Vivo PAM Fluorescence

**Fig. 4:**
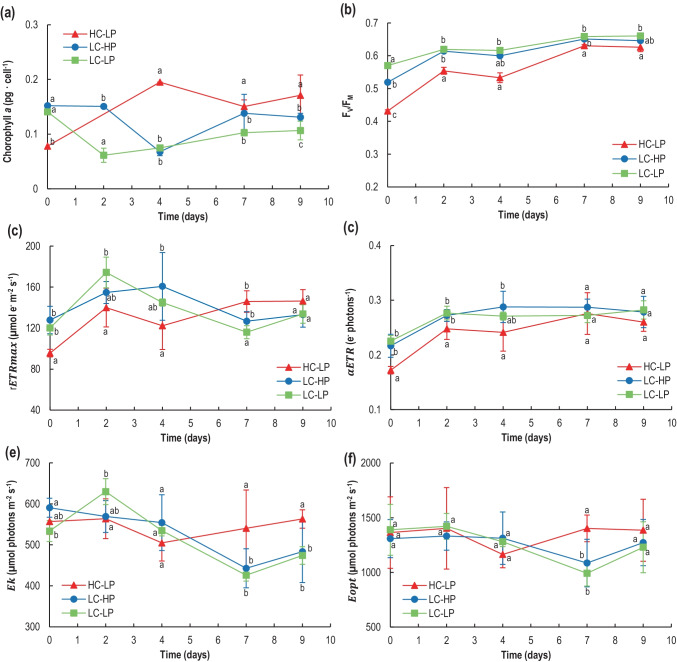
Photosynthetic parameters of rapid light curves in *E. huxleyi* cultures under conditions of HC-LP (1000–1200 µatm and pH 7.6–7.8), LC-HP (380–390 µatm and pH 8.2), and LC-LP (380–390 µatm and pH 7.6–7.8). Chlorophyll *a* (pg cell^−1^) (**a**), optimal quantum yield of Chl *a* associated to photosystem II (*F*_V_/*F*_m_) (**b**), relative maximum ETR (*rETR*_max_) (**c**), photosynthetic efficiency (*α*_ETR_) (**d**), saturation irradiance (*E*_k_) (**e**), and the highest irradiance just before photoinhibition occurs (*E*_opt_) (**f**). Values are mean ± SD (*n* = 3). Significant differences between treatments are indicated by different letters for any given time (*p* < 0.05)

Chlorophyll *a* per cell showed an initial decrease in all treatments up to d_2_–d_4_ (probably due to increased radiation reaching the cells after dilution at time d_3_), then it sharply increased, especially in high CO_2_ with respect to control treatment (Fig. 4a). Chl *a* cellular concentration was steady from d_7_ onwards, and it was similar to initial values in both LC treatments; however, in HC, it remained significantly higher. Initial *F*_V_/*F*_m_ values (Fig. 4b) ranged from 0.43 (high CO_2_) to 0.57 (low pH), and in all cases increased from the beginning to the end of the experiment, reflecting a temporary dilution stress and subsequent recovery. Final values around 0.63–0.66 were found in all treatments. However, *F*_V_/*F*_m_ was always significantly lower in high-CO_2_ cultures (*p* = 0.017, Sidak), suggesting not only that the photosynthetic performance was affected when aerated with high-CO_2_-enriched air, but also that acclimation to those conditions was possible, but slow. Both control and low-pH treatments exhibited a similar trend, without significant differences between treatments over time. The results of the low-pH compared to high-CO_2_ treatments indicate that the responses of the photosynthetic activity and the state of the photosystem II depend on the method used for acidification, with a negative effect by high CO_2_.

RLCs investigating photosynthetic performance indicated a similar maximum electron transport rate (*rETR*_max_) in all conditions for *E. huxleyi* cells (d_9_), albeit it was higher in both control and low-pH treatments during the first 4 days (Fig. 4c). The photosynthetic efficiency (α_ETR_) increased in all treatments as cultures developed, and at d_9_, there were no significant differences between treatments. The irradiance at which photosynthetic linear electron transport was saturated (*E*_k_) declined during cultivation in both control and low-pH treatments as cell density raised, but it remained high in high CO_2_ (Fig. 4e). This is in agreement with the lower cell density in this latter treatment (Fig. 2), reflecting the higher irradiance levels inside the cultures. Treatments had only marginal effects on the irradiance at which chronic photoinhibition is established (typically between 1200 and 1500 µmol quanta m^−2^ s^−1^), according to the *E*_opt_ values (Fig. 4f).

### Carbon Fixation Performance

**Fig. 5:**
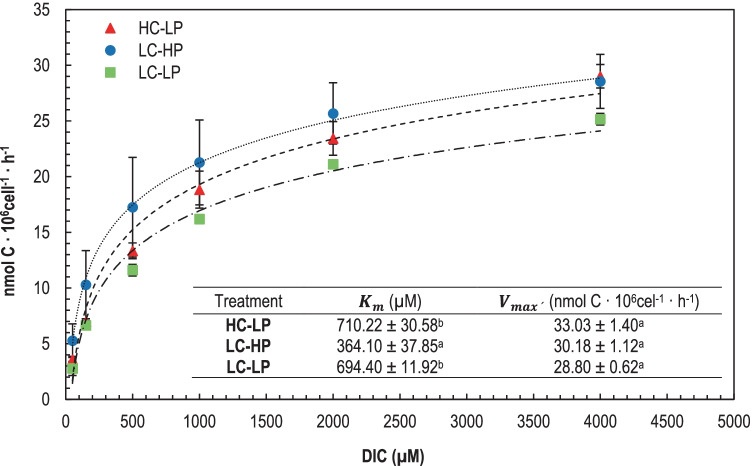
Carbon fixation rate showing *K*_M_ (µM) and *V*_max_ (nmol C · 10^6^ cell^−1^ · h^−1^) for *E. huxleyi* in d_4_ under HC-LP (1000–1200 µatm and pH 7.6–7.8), LC-HP (380–390 µatm and pH 8.1), and LC-LP (380–390 µatm and pH 7.6–7.8) treatments. DIC concentrations in the assay medium were 50, 150, 500, 1000, 2000, and 4000 µM. The kinetic parameters were calculated by fitting to Michaelis–Menten kinetics. Values are mean ± SD (*n* = 3). Significant differences between treatments are indicated by different letters (*p* < 0.05)

A typical Michaelis–Menten saturation kinetic resulted from measuring C fixation at increasing DIC concentrations in cells from all three treatments (Fig. 5). *V*_max_ was not significantly different between the treatments. Values varied between 28.80 ± 0.62 and 33 ± 1.40 nmol C 10^6^ cell^−1^ h^−1^, revealing similar RuBisCO fixation ability for all culture conditions (*p* < 0.05, Table S5). However, both acidification conditions (HC-LP and LC-LP) produced a significant decrease in DIC affinity according to nearly double *K*_M_ values with respect to the control (LC-HP). This result indicates that carbon uptake was negatively affected by low pH rather than high CO_2_.

### pH Drift and Stable δ^13^C Isotopic Determination

The pH compensation point is the final pH obtained in the pH drift experiment. This is the maximal pH value that the *E. huxleyi* cultures were able to reach while performing photosynthesis, as measured at the end of the culture period (day 9). It is commonly assumed that pH values greater than 9.0 pinpoint the ability of the cells to use HCO_3_^−^, in addition to CO_2_, as a source of inorganic carbon for photosynthesis. This capacity was observed under control conditions (final pH 9.7). However, both low-pH treatments decreased the ability of *E. huxleyi* to use HCO_3_^−^, specifically when acidification was caused by high pCO_2_ aeration (HC-LP; Table 1). The isotopic discrimination values (δ^13^C_microalgae_) in *E. huxleyi* (Table 1) were significantly more negative in high-CO_2_ cultures, decreasing from − 16.1‰ for LC-HP (control) to − 28.4‰ for HC-LP (*p* < 0.05, Table S5). These data are close to the theoretical limit of − 30‰, indicative of downregulation of CCMs; however, this interpretation must be taken with caution as it is determined by the specific discrimination of RuBisCO. Boller et al. [57] gave an unexpected value for RuBisCO discrimination of − 11‰. Wilkes and Pearson [58] suggest a mechanism by which the in vitro carbon isotope discrimination of RuBisCO in Boller et al. [57] can be reconciled with in vivo carbon isotope discrimination of *E. huxleyi*. Nonetheless, when acidification was produced by HCl addition, δ^13^C_microalgae_ decreased only slightly, to − 18.2 ‰, evidencing the requirement for high concentrations of CO_2_ to deactivate CCMs in *E. huxleyi*.

### Calcification and Morphometry

**Fig. 6:**
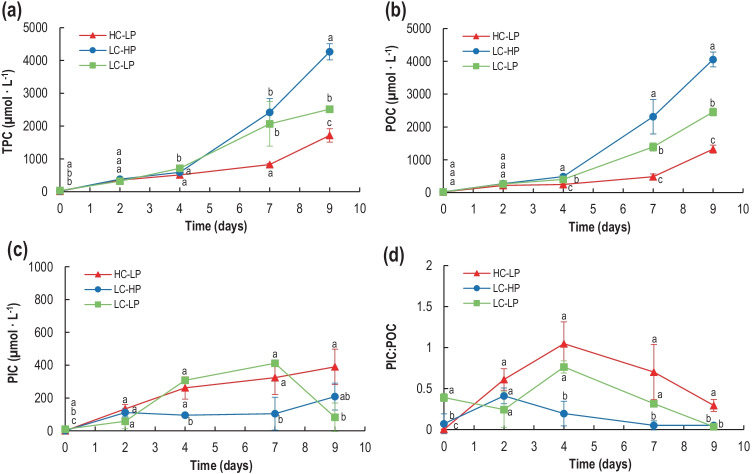
Temporal development of total particulate carbon, TPC (**a**), particulate organic carbon, POC (**b**), and particulate inorganic carbon, PIC (**c**) concentrations (µmol L^−1^) and molar inorganic C (PIC): organic C ratio (POC) (**d**) in *E. huxleyi* cultures under HC-LP (1000–1200 µatm and pH 7.6–7.8), LC-HP (380–390 µatm and pH 8.2), and LC-LP (380–390 µatm and pH 7.6–7.8). Values are mean ± SD (*n* = 3). Significant differences between treatments are indicated by different letters for any given time (*p* < 0.05)

TPC as well as POC in the cultures rapidly increased after cell inoculation in all treatments (Fig. 6a, b). Up to day 4, there were no significant differences between treatments (Table S4). However, from d_4_ onwards, significant differences were found. The highest concentrations of both TPC and POC (µmol L^−1^) occurred in control cultures (LC-HP), while they reached just one-third of the control concentration at high CO_2_. The low-pH treatment showed an intermediate trend. These data partly reflected the growth of *E. huxleyi* in the three conditions tested in this work. A different scenario was found for PIC. In the control cultures, PIC increased during the first 2 days, remaining steady to the end of the experiment (Fig. 6c). PIC highest accumulation occurred at d_9_ in high CO_2_ and at d_7_ in low pH. It returned to initial values at the end of the experiment. Ratio of PIC:POC transiently increased in all treatments during d_2_ to d_4_, dropping to initial values after at d_9_. PIC:POC was always higher in high CO_2_, reflecting the accumulation of PIC in the medium, concomitant with a lower POC. In both control and low-pH conditions, PIC represented a very small fraction of the total particulate carbon in the cultures.

POC and PIC per cell sharply increased in all treatments during the first 4 days, coincident with the lag phase of growth. This increase was more pronounced in high-CO_2_ conditions (HC-LP) (Table 2). POC further decayed by 50–75% in high CO_2_ as compared to the other two treatments. The similar trend was found for cell PIC content but, at the end of the experimental period, PIC was 7- to 15-fold higher at high-CO_2_ cultures. These data indicate that on a per cell basis, *E. huxleyi* accumulated more POC and PIC in high carbon compared to low carbon conditions.

**Table 2:** Particulate organic (POC) and inorganic (PIC) carbon cell quotas for *E. huxleyi* (pg cell^−1^) in HC-LP (1000–1200 µatm and pH 7.6–7.8), LC-HP (380–390 µatm and pH 8.1), and LC-LP (380–390 µatm and pH 7.6–7.8). Values are mean ± SD (*n* = 3). Significant differences between treatments are indicated by different letters for any given time (*p* < 0.05)

Day	POC (pg cell^−1^)			PIC (pg cell^−1^)		
	HC-LP	LC-HP	LC-LP	HC-LP	LC-HP	LC-LP
0	23.61 ± 2.83^a^	25.82 ± 9.40^a^	29.40 ± 0.91^a^	0.12 ± 0.00^a^	3.62 ± 0.00^b^	11.47 ± 1.60^c^
2	285.87 ± 23.91^a^	97.76 ± 0.48^b^	81.31 ± 11.42^b^	174.17 ± 35.93^a^	40.16 ± 9.47^b^	27.31 ± 9.75^b^
4	241.51 ± 7.28^a^	23.83 ± 0.23^b^	26.45 ± 1.93^b^	253.24 ± 66.24^a^	4.66 ± 0.29^b^	20.09 ± 0.70^b^
7	31.36 ± 5.53^a^	19.18 ± 4.37^b^	16.50 ± 1.47^b^	21.06 ± 6.68^a^	0.86 ± 0.83^b^	4.89 ± 0.00^b^
9	35.68 ± 3.30^a^	23.70 ± 1.32^b^	20.65 ± 1.00^b^	10.50 ± 2.90^a^	1.49 ± 0.004^b^	0.69 ± 0.73^b^

**Fig. 7:**
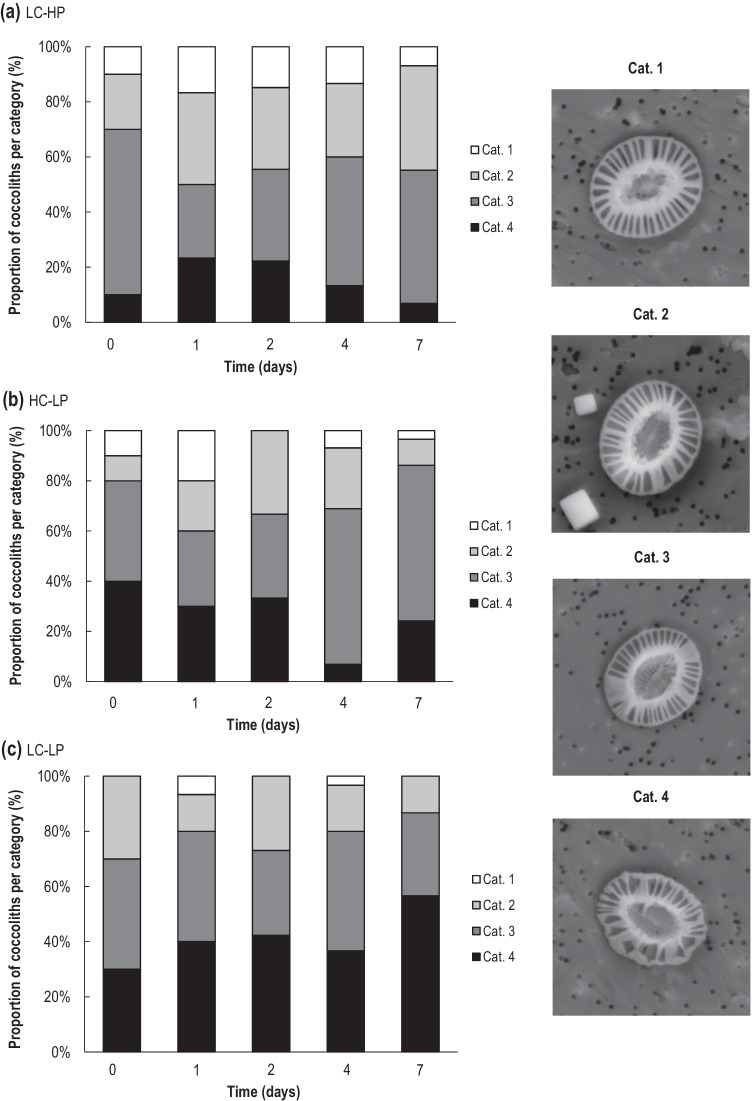
Percentage of *E. huxleyi* coccoliths per category. We assigned Cat. 1 to normal intact coccoliths, and Cat. 4 to fragmented coccoliths in all different treatments: LC-HP (380–390 µatm and pH 8.2) (**a**), HC-LP (1000–1200 µatm and pH 7.6–7.8) (**b**), and LC-LP (380–390 µatm and pH 7.6–7.8)

SEM images confirmed the presence of a single *E. huxleyi* morphotype in the cultures; coccospheres with heavily calcified elements and almost closed coccolith central area, corresponding to type A “overcalcified” (see Supplementary Fig. S1, and Supplementary Table S2) [59]. The morphological analysis revealed several degrees of coccolith malformation (Fig. 7) in the cultures throughout the experiment. The percentage of non-distorted coccoliths (Cat. 1) was higher in the high-CO_2_ and control treatments, with up to 20% and 16.67% respectively in d_1_, while only 6.67% of coccoliths showed no evidence of malformation in low pH at the same time. On the contrary, up to 56.67% (d_7_) of malformed coccoliths (Cat. 4) was observed in low pH, followed by 40% in d_0_ at high CO_2_. The highest percentage of 23.33% severely damaged coccoliths was found in d_1_, being lower in LC-HP. In general, the proportion of intact coccoliths decreased throughout the experiment in all treatments (Fig. 7). Similarly, the percentage of damaged coccoliths (Cat. 4) decreased over time in the control treatment while it increased in low pH. The proportion of severe malformation in coccoliths showed no significant relationship with carbonate chemistry variables. Detailed size morphology measurements of the coccoliths are shown in Supplementary Table S3.

## Discussion

The results indicate that the strain of *E. huxleyi* used in this study responded differently to acidification depending on whether it was caused by high levels of dissolved CO_2_ or by HCl addition. Several studies have previously focused on the differential effects of the acidification methods on several species of calcifiers and a varied strain of *E. huxleyi*, i.e., by means of increasing pCO_2_ maintaining TA constant, or by manipulating pH with concomitant change on TA [29], and diverse response patterns were observed. It is now known that the different strains of *E. huxleyi* show phenotypic plasticity regarding their growth performance, light-responses, calcification, and virus susceptibility [14]. This phenomenon is most likely a consequence of genomic differences, transcriptomic [14], and metabolomic [15] responses to environmental conditions, and/or threats such as viral infections [60]. Moreover, methodological differences are also responsible for the variety of response patterns observed in growth and photosynthetic performance, as well as in calcification [24, 29]. One of the methodological controversies when differentiating CO_2_ and pH effects as acidification methods is the aeration of the cultures. Shi et al. [61] compared responses of growth, POC and PIC quotas, and primary production of one *E. huxleyi* strain (NZEH), in closed TA and open DIC manipulations. Even though the cells responded differently under the two manipulations, it is still unclear whether this was due to differences in the carbonate chemistry or to mechanical effects of gas bubbles during aeration (independently of TA and DIC levels). The latter only occurred in the open DIC manipulation treatment and affected cell sedimentation. This was not the case in our study, since aeration was the same for all treatments and, additionally, mixing was ensured by gentle stirring at the bottom of the culture cylinders, rendering optimal growth rates and calcification in control conditions. Thus, the differences observed must be due to bubbling mechanical effects as previously demonstrated in this species [29].

Factors that affect growth rate and *F*_V_/*F*_m_ are likely to also affect the acclimation rate. For instance, in experiments that were carried out at a temperature significantly colder (8 °C) than the temperature used in our study (16 °C), about 10 days were needed for cells to be completely acclimated to the high-CO_2_ levels [62, 63]. However, studies at higher temperatures (22 °C) have shown that acclimation is normally reached 3–4 days after the start of the CO_2_ aeration [64]. Therefore, 72 h are well within the range to allow for full acclimation to the new pCO_2_ according to the temperatures used in our study.

Growth rate is a comprehensive variable integrating all physiological processes in marine phytoplankton, and its different responses to increased pCO_2_ have been reported for different strains of *E. huxleyi*, with positive, negative, or even no significant changes (e.g., [29] and references therein).To our knowledge, a notably stronger detrimental effect of acidification by using CO_2_ enrichment (HC-LP treatment in our experiment) than by increasing [H^+^] alone (LC-LP treatment) has not been previously reported. Yet, growth and organic carbon production rates in *E. huxleyi* seemed to be adversely affected by the associated decrease in pH directly related to high-CO_2_ levels [30, 51]. A reduction of 10 to 35% in growth rates at high CO_2_ have been also described for this species [13, 29], and the latter study [29] reports low sensitivity of growth to modifications of pH alone.

Importantly, a comparable effect with the present results has been reported for *C. leptoporus* [65] where adverse effects were caused by CO_2_ instead of pH. We discuss here the differences between the effects of the HC-LP and the LC-LP treatments despite the steady pH values, concluding that the differences lie on both DIC and CO_2_. Therefore, the different responses of the cells could be caused by either DIC or CO_2_, or indeed by both, most likely through increasing the CO_2_ concentration in the diffusive boundary layer of the cell [65]. Fukuda et al. [66] used experimental pH and CO_2_ treatment levels similar to the one used in the present study. The strain they used was originally from the Pacific Ocean (NIES 837) and their results showed an opposite trait for growth than ours, i.e., growth increased at high CO_2_, and decreased at lower pH (without CO_2_ enrichment). They did not bubble continuously with CO_2_-enriched air, which suggest that those results are to be taken carefully.

Lorenzo et al. [21] used the same strain as in the present study but cultured cells at 400 and 800 µatm CO_2_ and found no significant effect of increased CO_2_ on the growth rate. Two reasons account for the apparent contradiction with the results shown here: (1) they used the exponential model of growth to calculate the growth rate instead of the logistic model, which resulted in an underestimation; and (2) their highest CO_2_ concentration was notably lower than the one used in the present work (1200 µatm). Hoppe et al. [29] also reported invariable growth rates up to 800 µatm, declining significantly beyond this value (highest CO_2_ concentration tested 1200 µatm). Similarly, Bach et al. [30] did not find significant differences in a smaller range up to 600 µatm. This supports the idea that detrimental effects of CO_2_ on growth rates occur at CO_2_ levels above 800 ppm. Nevertheless, other reports have shown negative effects of CO_2_ on growth rate at 600 ppm [13]. Since it is expected that by the end of this century the level of atmospheric CO_2_ will be over 900 ppm, *E. huxleyi* is predicted to be negatively affected in the near future.

Growth rates at high-CO_2_ and control conditions in this experiment were very similar to those found by Segovia et al. [20] under similar treatments (but using around 1000 µatm as high-CO_2_ level) in a mesocosm experiment in the Norwegian coast with a natural population. This suggests that both the natural population growing in those mesocosms and the strain used here had similar CO_2_ sensitivity and growth performance. These authors also showed, as here, a strong restriction of the maximum cell density at high CO_2_. The presence of high levels of CO_2_ did not only prevent cell division but also promoted the unviability of already formed cells [23]. It also affected the cellular level of many metabolites (175 out of 333 metabolites identified, [15]). All aminoacids (except glycine) and all detected TCA cycle substrates decreased at high CO_2_ relative to control conditions. Our results also indicate a metabolic misbalance (i.e., changes that affected the allocation of carbon and cellular energy efficiency) induced by high CO_2_, but not by lowering pH alone. Decreased cell viability promoted by high-CO_2_ levels was also reported for the above-mentioned Norwegian natural population mesocosm experiment [23]. It is known that elevated CO_2_ can impair signal transduction as well as ion-transport and catabolic processes [31]; yet, to our knowledge, this is the first study to directly point to CO_2_, and not acidification, as the cause of such impediments. A possible explanation is the permeation of CO_2_ to the intracellular space and its spontaneous conversion to HCO_3_^−^ and H^+^, the latter deterring the processes mentioned above. Indeed, Langer and Bode [65] suggested that intracellular acidification through increased CO_2_ might be due to the diffusive boundary mechanism. Blanco-Almeijeiras et al. [67] demonstrated that the plasma membrane of *E. huxleyi* was permeable to CO_2_ but nearly impermeable to HCO_3_^−^ under a high-CO_2_ environment, supporting the variation in carbon isotopic fractionation of photosynthetically produced organic matter assuming solely diffusive acquisition of CO_2_. In contrast, Suffrian et al. [68] are not able to detect a CO_2_ permeability high enough to change intracellular pH.

The onset of the cultures seemed to have imposed some sort of stress on the cells, probably due to increased irradiance after dilution of the stock culture, according to their relatively high signal for ROS accumulation and low *F*_V_/*F*_m_. While cells under normal CO_2_ conditions were able to detoxify the high ROS as the cultures progressed, cells under high-CO_2_ conditions remained with high levels of stress. Genes encoding for ROS scavenging antioxidants, enzymes the synthesis of vitamin B_6_ mediating photo-oxidative stress in plants, and many light-harvesting complex (LHC) proteins have also been found in the core genome of *E. huxleyi* [14] and, more interestingly, they have been reported to orchestrate the response to stress in the coccolithophore under high pCO_2_ [23]. At least, some of these processes seemed to be also hindered by high-CO_2_ conditions, but not by low pH.

CO_2_-dependent changes in photosynthesis are highly variable and seem to differ between strains. Lorenzo et al. [21] found no differences in *ETR*_max_ between 400 and 800 ppm CO_2_ using the same strain. Also, Segovia et al. [23] found very discrete changes in fluorescence-derived photosynthetic parameters in a natural *E. huxleyi* population from Norway. Fukuda et al. [66], using a Pacific strain, reported no difference in *F*_V_/*F*_m_ and effective quantum yield (inversely related to ETR), either under acidification alone or with extra pCO_2_. It is common that CO_2_ uncouples photosynthetic C production from growth [33]. Growth restriction without effect on photosynthesis usually leads to organic C accumulation in the cell [33]. In this study, POC per cell was highest in HC cultures, particularly in the first half of the culture period, when most of the cells were still viable. Yet, cells were considerably smaller when a decrease in cell viability occurred over time. This evidences that the fate of C can vary depending on the growth phase of the culture, even when the photosynthetic capacity remains constant, as discussed below.

In other phytoplankton species, high-CO_2_ levels enhanced growth and photosynthesis protecting against photoinhibition [69]. In this sense, increased CO_2_ would have a positive role in photoprotection [69]. However*, E. huxleyi* presents a remarkable capacity to withstand photoinhibition even in normal CO_2_ conditions [12]. The coccolithophore’s core genome encodes a variety of photoreceptors, and related proteins that function in the assembly and repair of photosystem II, such as D1-specific proteases and FtsH enzymes, as well as proteins that have a role in non-photochemical quenching (NPQ) or synthesis of NPQ compounds [14]. The complex repertoire of such photoprotectors facilitates tolerance to high light minimizing ROS accumulation and preventing oxidative damage, so that, presumably, increased CO_2_ would not pose a positive selection pressure on *E. huxleyi* populations at the photochemistry level.

Carbon uptake by *E. huxleyi* is influenced by the pH of the assay medium and by the resulting carbonate chemistry, rather than by the pCO_2_ condition during acclimation [33]. However, since our ^14^C-based method for the determination of C uptake kinetics was performed in a buffered medium (pH 8) in all cases, the differences can only be attributed to culture conditions and not to the assay conditions. The decrease in C_i_ affinity (higher *K*_M_) can be ascribed to partial deactivation of some of the components of the CCMs. Like most phytoplankton, *E. huxleyi* operates a CCM which accumulates CO_2_ in the vicinity of RuBisCO [70, 71]. The deactivation of the CCM is corroborated by lower (more negative) values of δ^13^C_microalgae_ and lower pH compensation points in the high-CO_2_ cultures but not in low-pH ones. Hence, the decreased affinity, at least in the latter, might be due to a weaker H^+^ gradient across the plasma membrane that could provoke a CO_2_ leakage from within the cell. Leakage of CO_2_ was higher when the CO_2_ gradient between the cytosol and the external medium increased [72]. In our experiment, this CO_2_ gradient was most likely larger at low external pCO_2_, and the loss of CO_2_ via leakage (and therefore the reduction in carbon fixation affinity) could have been more pronounced under these conditions (LC cultures). The leakage of CO_2_ strongly increased at pCO_2_ levels below 200 µatm in several other species [73, 74]. However, its effect on C fixation can be reverted by an active HCO_3_^−^ transporter. *E. huxleyi* relies mainly on CO_2_ diffusive entry at pH < 8.1 [33] (as in LC-LP, Table 1), but can use a HCO_3_^−^ transporter at higher pH (as in the control). By using the isotope disequilibrium assay [70], it was demonstrated that the *E. huxleyi* strain studied in a natural phytoplankton community from coastal waters of Norway did use HCO_3_^−^ transporters actively, so that the main *C*_i_ source was HCO_3_^−^ [71]. This could account for the difference in *C*_i_ affinity and CCM activation level of the three treatments used in this work.

Since the response of ocean chemistry to increasing pCO_2_ involves the decrease in calcium carbonate saturation that might affect biological calcification, many acidification experiments commonly focus on calcification by coccolithophores. Such experiments have generally shown a negligible to relatively large decrease in calcification at high pCO_2_, but there are also reports on higher calcification at high CO_2_/low pH, depending on the species, strains, and methodology used [75]. PIC and POC content per cell indicated an over-accumulation with respect to the control (LC-HP) during the exponential growth phase, which was much more obvious at high CO_2_. An increase in the cellular POC quotas at higher CO_2_/lower pH is more commonly reported than the increase in PIC quotas. Usually, calcification is defective under corrosive conditions, and rapid coccolith dissolution has been observed to start with Ω_calcite_ values below 0.4 [30]. We did not obtain Ω_calcite_ values below 0.4 at high CO_2_, but we did at low pH. Malformed coccospheres were more abundant under both acidified conditions, but particularly in the low-pH treatment. Thus, increased CO_2_ did not restrict calcification; on the contrary, both POC and PIC accumulation in high-CO_2_-grown cells seems to have served as a fate of C_i_ being taken up and not used for growth. The LC-LP treatment was characterized by a substantial dissolution. Data supports this inference because while the cultures were still growing, the PIC quota (both cell and volume normalized) was decreasing. In parallel, the DIC and TA decrease leveled off. Taken together, these results strongly support PIC dissolution. SEM analyses showing an increased level of Cat. 4 “malformations” in LC-LP (Fig. 7) reinforce the former affirmation on PIC dissolution. Cat. 4 morphology is possibly a malformation but could equally possibly be a dissolution-morphology. In *E. huxleyi*, malformation and dissolution can be very difficult to differentiate. It is not possible to assess PIC production in this treatment and that makes complex to interpret coccolith morphology because Cat. 4 coccoliths here are likely a mix of malformed and partially dissolved ones. PIC and POC cellular quotas in the present work varied notably during the different phases of the culture. This means that experiments in which samples are either taken at a specific time point or kept in semi-continuous exponential phase of growth do not describe completely the overall behaviour of an *E. huxleyi* bloom. In this sense, the high PIC and POC values observed perfectly correlated with the long lag phase of growth found under those conditions. Cells did not start to divide in HC-LP until approx. day 4, and we presume that accumulation of both PIC and POC was occurring. When active growth resumed, cell POC and PIC quotas dropped off to normal values. Thus, we think that this overcalcified type A strain might have had cell division arrested during that phase of the experiment while photosynthesis and calcification were active, hence accumulating cellular POC and PIC. Clearly, these are exceptional values that have never been reported before. We also demonstrate in this study that other relevant variables such as cell viability, ROS accumulation, and PIC:POC ratio depend on the phase of the bloom, as also previously reported in mesocosm experiments [23, 71].

As concluding remarks, the results obtained with the strain of *E. huxleyi* used here highlight the capacity of CO_2_ rather than acidification itself to generate metabolic stress and functional imbalance (meaning metabolic changes that affected the allocation of carbon and cellular energy efficiency). The metabolic and growth impairment caused by high CO_2_ exceeded the small effect on photochemistry, even though a considerable accumulation of ROS was recorded. The precise mechanism by which CO_2_ permeating the cell (and/or enhanced CO_2_ leakage) may interfere with the metabolism of *E. huxleyi* still remains to be elucidated. Accordingly, the levels of CO_2_ expected in the near future may compromise growth and cell viability (despite the small effect on calcification). CO_2_ perturbation experiments are major tools used to mimic future ocean scenarios and to study the response of relevant organisms contributing to the C cycle and the biological pump. Data supply for large-scale system models critically depend on ecophysiological studies of functional groups such as calcifying organisms. The diversity of the response depicted here reflects the difficulty to estimate the amount of anthropogenic CO_2_ taken up by globally important calcifying species such as *E. huxleyi.* The heterogeneous behaviour of this species indicates that a single strain is unlikely to represent the whole species. It is also worth noting that other environmental drivers (such as temperature and Fe availability) may be equally or more influential than CO_2_ and pH in regulating the physiological responses of *E. huxleyi*; hence, more multistressors experiments are needed to improve our understanding on how phytoplankton communities will develop in our future high-CO_2_ oceans.

## Supplementary Information

Below is the link to the electronic supplementary material.

Supplementary file1 (PDF 1923 KB)

Supplementary file2 (PDF 407 KB)

## Data Availability

All data generated or analysed during this study are included in this published article (and its Supplementary Information files).
