# Supplementary material for: High-CO2 Levels Rather than Acidification Restrict Emiliania huxleyi Growth and Performance
Source: Microb Ecol. 2022 May 27;86(1):127–43. doi: 10.1007/s00248-022-02035-3 (PMC10293452; doi:10.1007/s00248-022-02035-3)
Supplement: Supplementary file 1 — Supplementary file1 (PDF 1923 KB) [file 248_2022_2035_MOESM1_ESM.pdf]

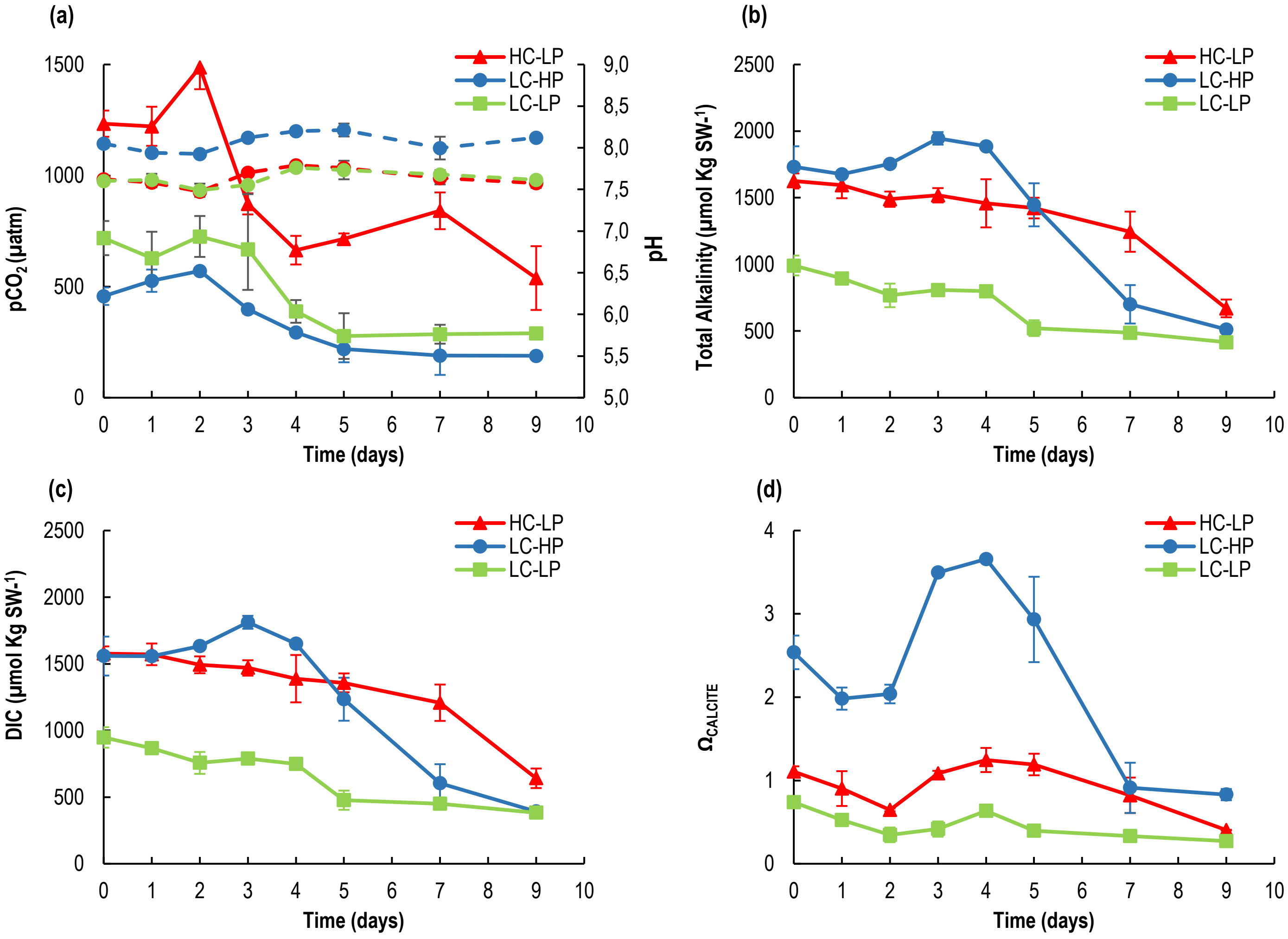

Figure 1. Measured pH (dotted line) (a) and total alkalinity (b), and calculated pCO_2 (solid line) (a), dissolved inorganic carbon (DIC) (c), and calcite saturation estate ($\Omega_{calcite}$) (d), in the different treatments: HC-LP (pCO_2: 1000-1200 µatm; pH: 7.6-7.8), LC-HP (pCO_2: 380-390 µatm; pH: 8.2) and LC-LP (pCO_2: 380-390 µatm; pH: 7.6-7.8). Values are mean ± SD (n = 3). Significant differences between treatments are indicated by different letters for any given time ($p < 0.05$).

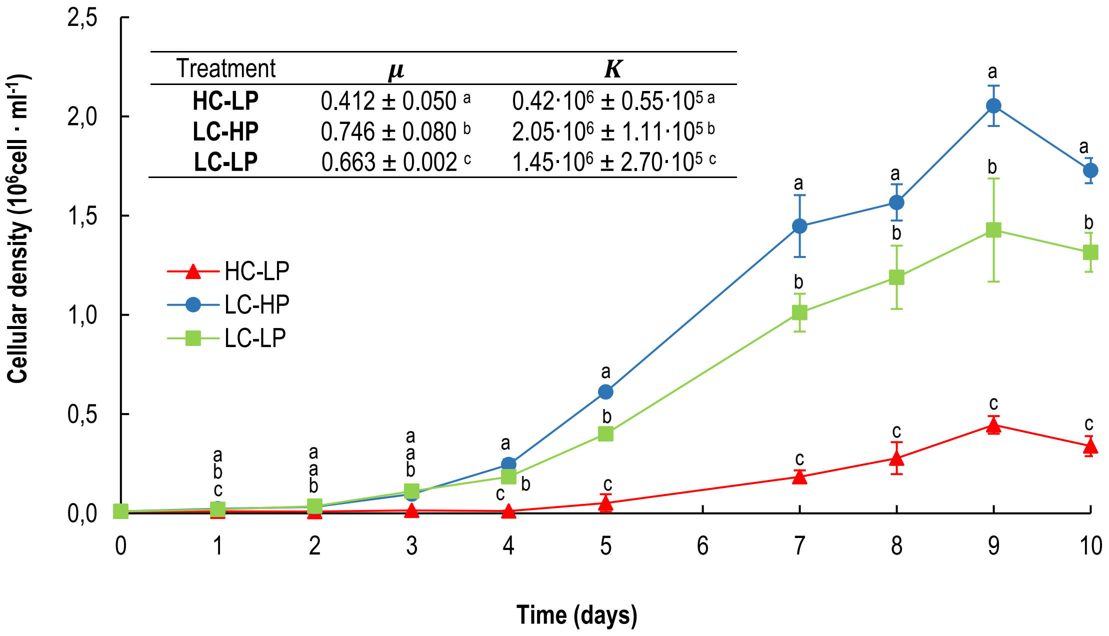

Figure 2. Cellular abundance (10^6cell ml^{-1}), net growth rates (μ, d^{-1}) and loading capacity (K, 10^6cell ml^{-1}) of *E. huxleyi* cultures calculated with the logistic model of growth under HC-LP (1000-1200 µatm and pH 7.6-7.8), LC-HP (380-390 µatm and pH 8.1) and LC-LP (380-390 µatm and pH 7.6-7.8). Values are mean ± SD (n = 3). Significant differences between treatments are indicated by different letters for any given time ($p < 0.05$).

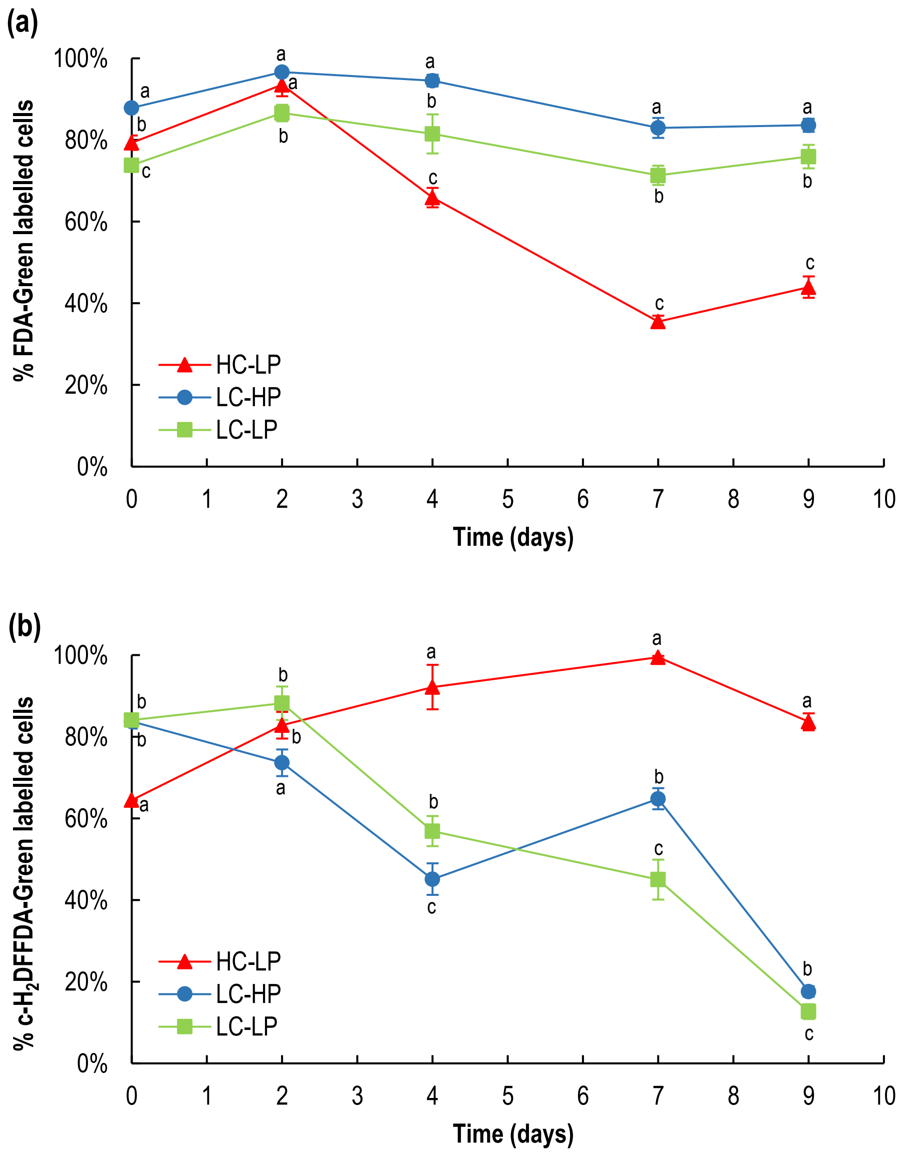

Figure 3. Variation of (a) cell viability measured as FDA-Green fluorescence labelled *E. huxleyi* cells and, (b) reactive oxygen species (ROS) measured as c-H_2DFFDA-Green fluorescence labelled *E. huxleyi* cells in HC-LP (1000-1200 µatm and pH 7.6-7.8), LC-HP (380-390 µatm and pH 8.1) and LC-LP (380-390 µatm and pH 7.6-7.8). Values are mean ± SD (n = 3). Significant differences between treatments are indicated by different letters for any given time ($p < 0.05$).

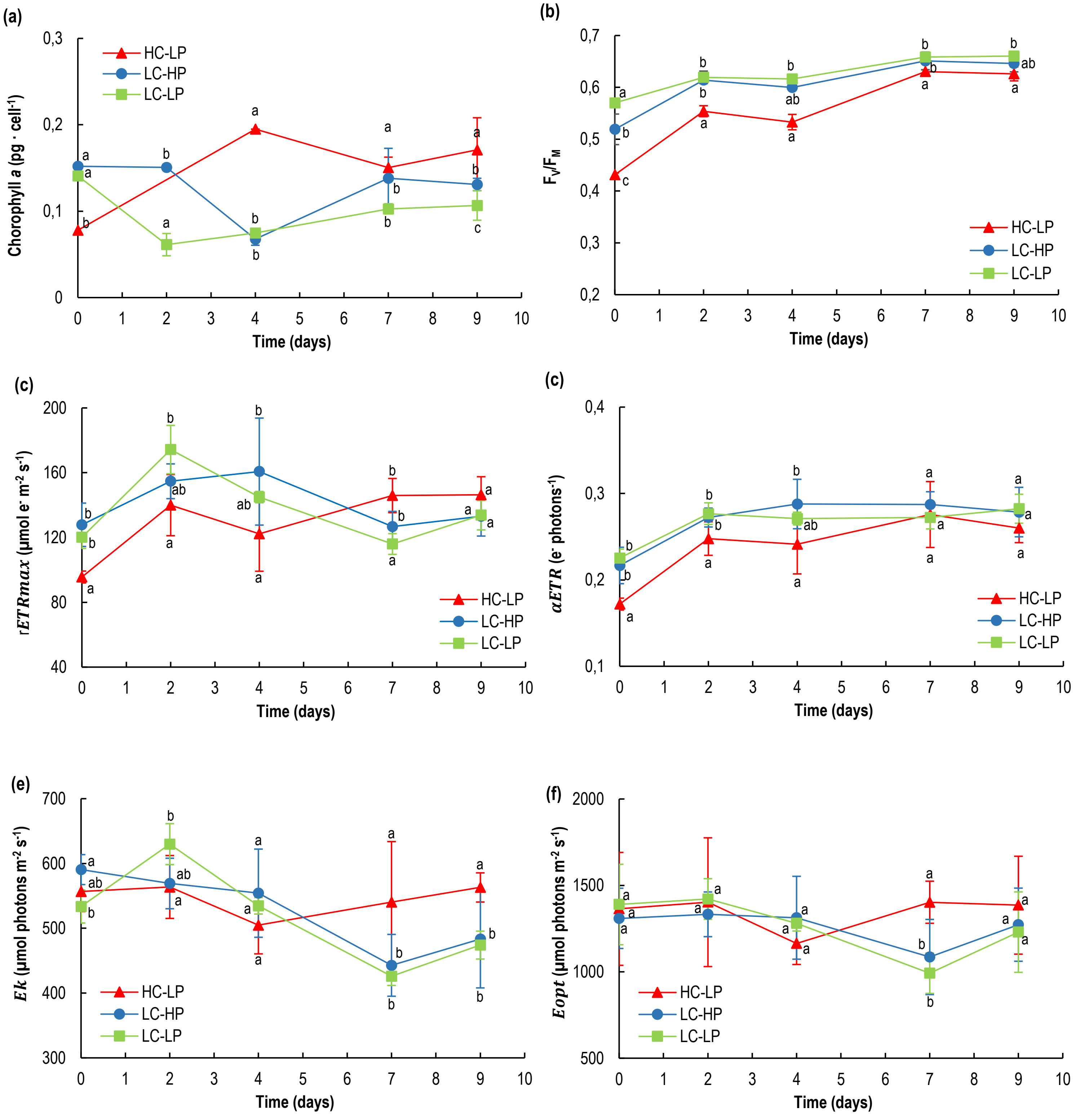

Figure 4. Photosynthetic parameters of rapid light curves in *E. huxleyi* cultures under conditions of HC-LP (1000-1200 µatm and pH 7.6-7.8), LC-HP (380-390 µatm and pH 8.2) and LC-LP (380-390 µatm and pH 7.6-7.8). Chlorophyll *a* (pg cell^{-1}) (a), optimal quantum yield of Chl *a* associated to photosystem II (F_V/F_M) (b), relative maximum ETR ($rETR_{max}$) (c), photosynthetic efficiency (α_{ETR}) (d), saturation irradiance (E_k) (e), and the highest irradiance just before photoinhibition occurs (E_{opt}) (f). Values are mean ± SD (n = 3). Significant differences between treatments are indicated by different letters for any given time ($p < 0.05$).

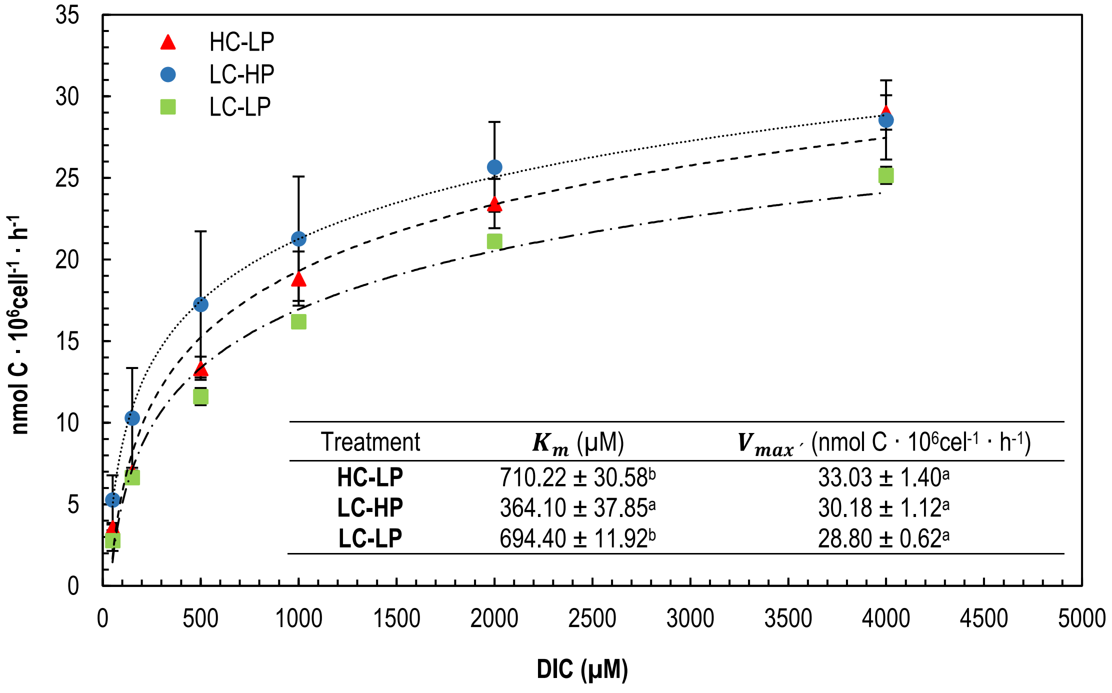

Figure 5. Carbon fixation rate showing K_m (µM) and V_{max} (nmol C · 10^6cel^{-1} · h^{-1}) for *E. huxleyi* in d_4 under HC-LP (1000-1200 µatm and pH 7.6-7.8), LC-HP (380-390 µatm and pH 8.1) and LC-LP (380-390 µatm and pH 7.6-7.8) treatments. DIC concentrations in the assay medium were 50, 150, 500, 1000, 2000, 4000 µM. The kinetic parameters were calculated by fitting to Michaelis-Menten kinetics. Values are mean ± SD (n = 3). Significant differences between treatments are indicated by different letters ($p < 0.05$).

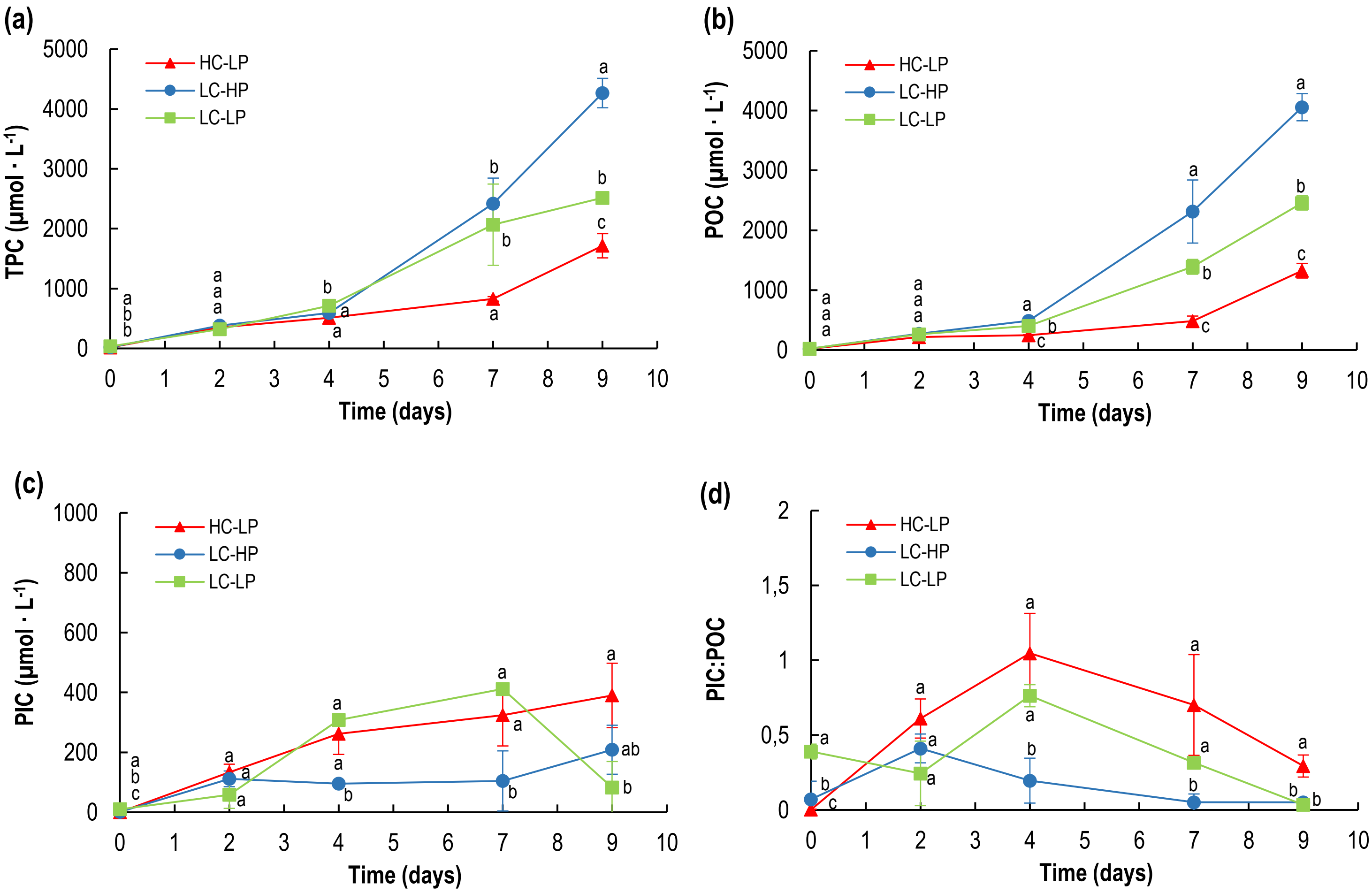

Figure 6. Temporal development of total particulate carbon, TPC (a), particulate organic carbon, POC (b) and particulate inorganic carbon, PIC (c) concentrations (µmol L^{-1}) and molar inorganic C (PIC): organic C ratio (POC) (d) in *E. huxleyi* cultures under HC-LP (1000-1200 µatm and pH 7.6-7.8), LC-HP (380-390 µatm and pH 8.2) and LC-LP (380-390 µatm and pH 7.6-7.8). Values are mean ± SD (n = 3). Significant differences between treatments are indicated by different letters for any given time ($p < 0.05$).

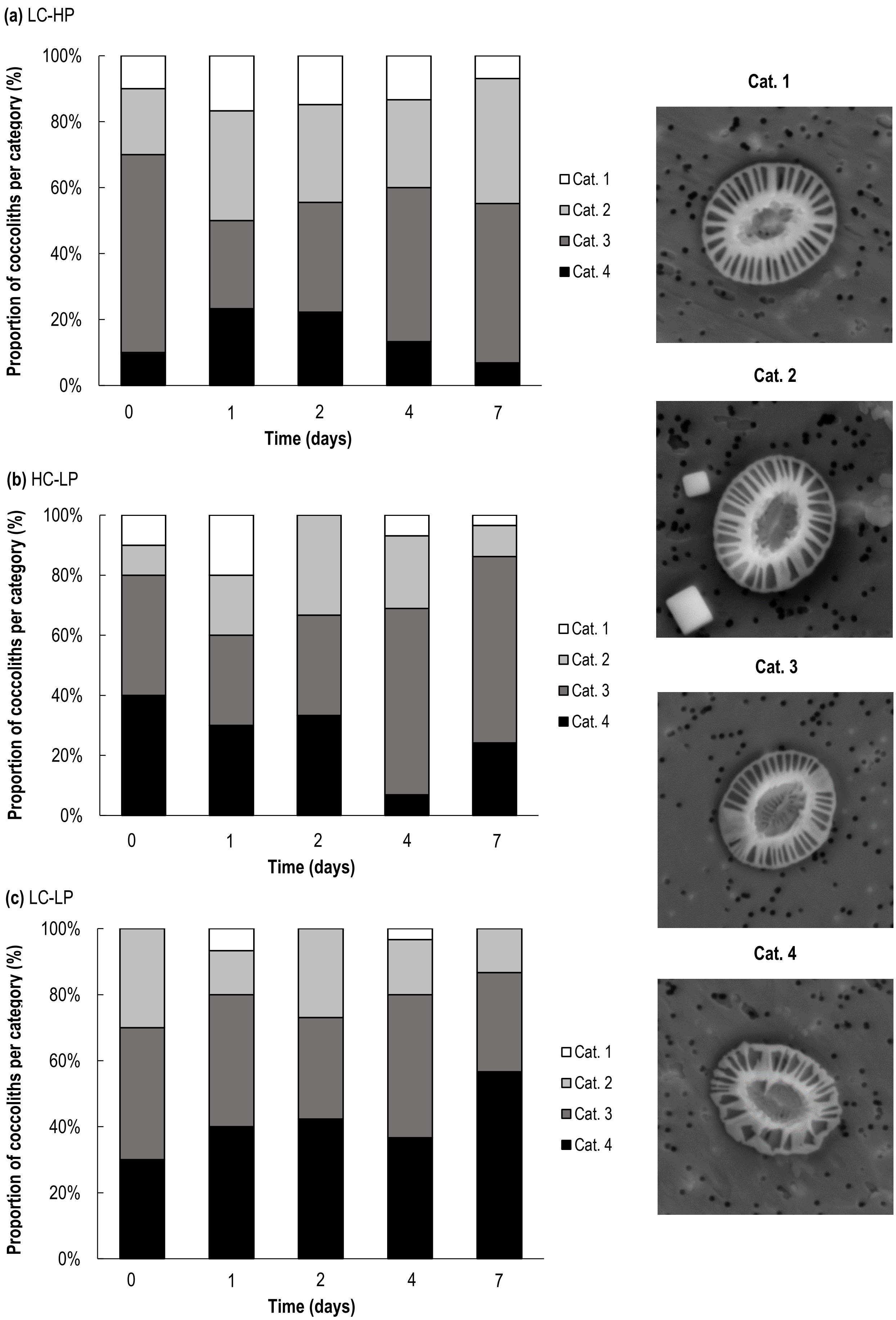
(a) LC-HP
(b) HC-LP
(c) LC-LP
Proportion of coccoliths per category (%)
0%
20%
40%
60%
80%
100%
0
1
2
4
7
Time (days)
Cat. 1
Cat. 2
Cat. 3
Cat. 4

Figure 7. Percentage of *E. huxleyi* coccoliths per category. We assigned cat. 1 to normal intact coccoliths, and cat. 4 to fragmented coccoliths in all different treatments; LC-HP (380-390 µatm and pH 8.2) (a), HC-LP(1000-1200 µatm and pH 7.6-7.8) (b) and LC-LP 380-390 µatm and pH 7.6-7.8).

(a) LC-HP

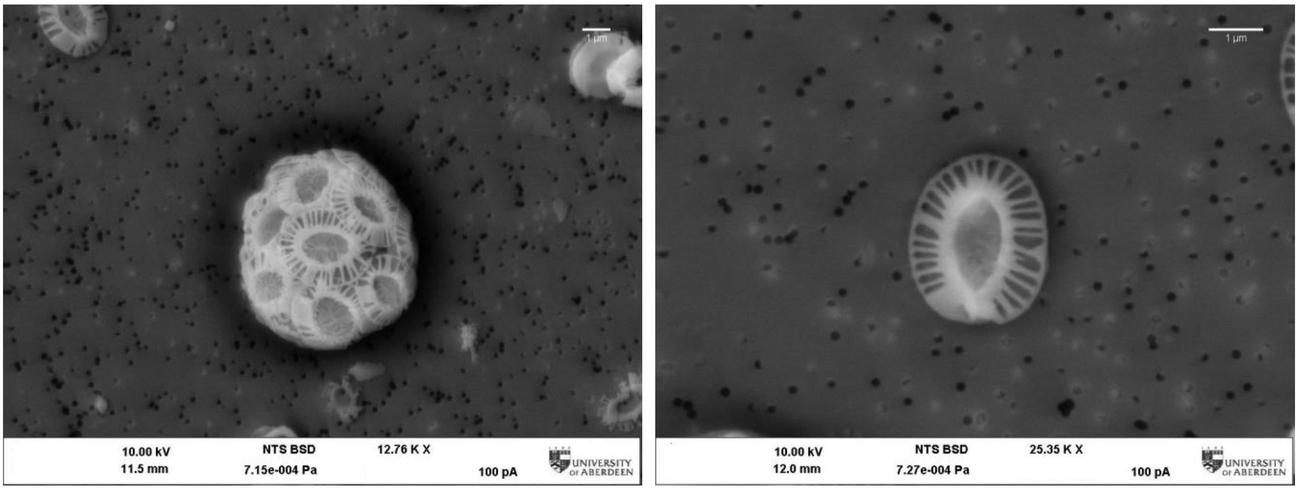

(b) HC-LP

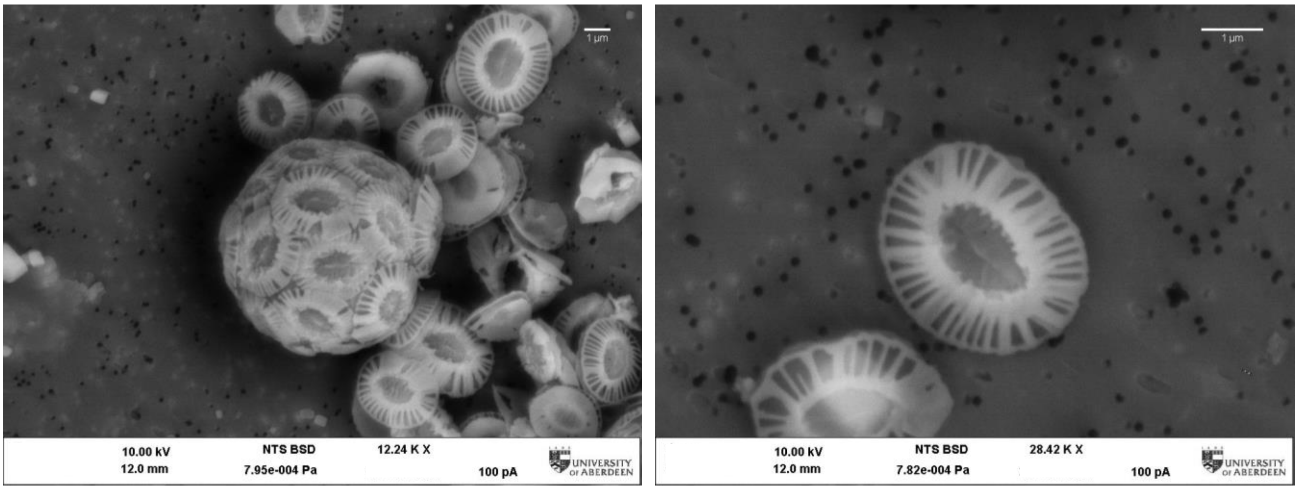

(c) LC-LP

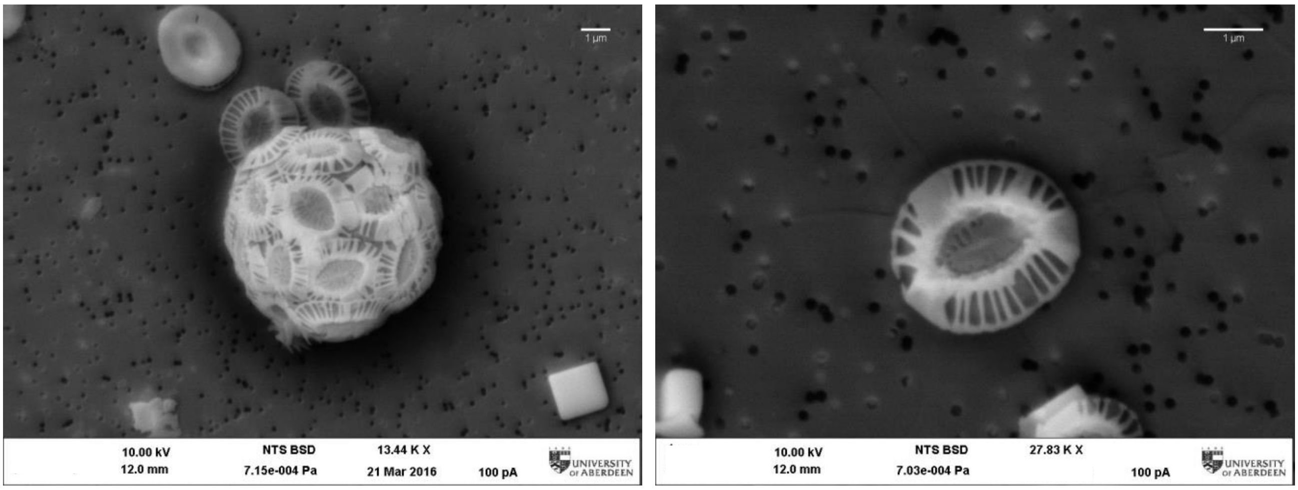

Figure S1. Representative scanning electron microscopy (SEM) images of *E. huxleyi* cells grown under different concentrations of CO_2 and pH values on d_4. (a) 380-390 µatm and pH 8.2; (b) 1000-1200 µatm and pH 7.6-7.8 and (c) 380-390 µatm and pH 7.6-7.8. The scale bars are in µm. The images were selected from a large dataset of SEM micrographs to show the general pattern in calcification of coccospheres and coccoliths.

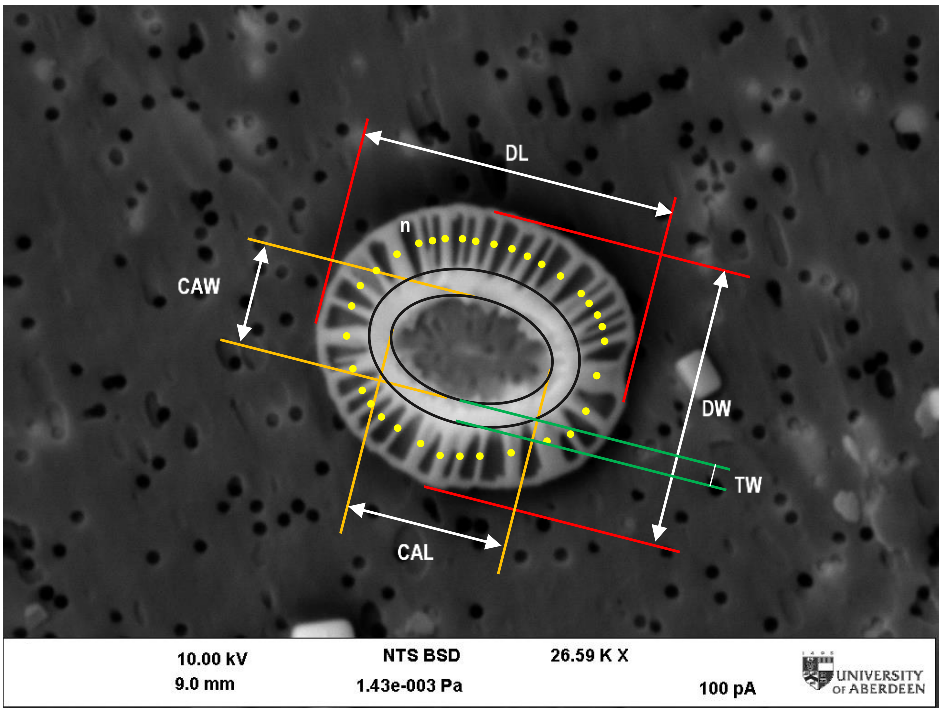

Figure S2. Morphometric parameters of coccoliths of *E. huxleyi* measured in distal view scanning electron micrographs (SEM). DL, Length of the distal shield; DW, width of the distal shield; CAL, length of the central area; CAW, width of the central area; n, number of segments; TW, tube width.

Treatment	$\boldsymbol{\delta^{13}C_{microalgae}}$ (‰)	pH compensation point
**HC-LP**	-28.37 ± 1.10[a]	8.02 ± 0.03[a]
**LC-HP**	-16.07 ± 0.27[b]	9.70 ± 0.07[b]
**LC-LP**	-18.17 ± 1.15[b]	9.17 ± 0.03[c]

Table 1. Corrected $\partial^{13}C$ isotopic discrimination ($\partial^{13}C_{microalga}$) and pH compensation point for *E. huxleyi* under the different experimental conditions on day 9; HC-LP (pCO_2: 1000-1200 µatm; pH: 7.6-7.8), LC-HP (pCO_2: 380-390 µatm; pH: 8.2) and LC-LP (pCO_2: 380-390 µatm; pH: 7.6-7.8). Values are mean ± SD. Significant differences between treatments are indicated by different letters ($p < 0.05$).

	**POC** (pg cell^{-1})			**PIC** (pg cell^{-1})		
**Day**	**HC-LP**	**LC-HP**	**LC-LP**	**HC-LP**	**LC-HP**	**LC-LP**
0	23.61 ± 2.83^{a}	25.82 ± 9.40^{a}	29.40 ± 0.91^{a}	0.12 ± 0.00^{a}	3.62 ± 0.00^{b}	11.47 ± 1.60^{c}
2	285.87 ± 23.91^{a}	97.76 ± 0.48^{b}	81.31 ± 11.42^{b}	174.17 ± 35.93^{a}	40.16 ± 9.47^{b}	27.31 ± 9.75^{b}
4	241.51 ± 7.28^{a}	23.83 ± 0.23^{b}	26.45 ± 1.93^{b}	253.24 ± 66.24^{a}	4.66 ± 0.29^{b}	20.09 ± 0.70^{b}
7	31.36 ± 5.53^{a}	19.18 ± 4.37^{b}	16.50 ± 1.47^{b}	21.06 ± 6.68^{a}	0.86 ± 0.83^{b}	4.89 ± 0.00^{b}
9	35.68 ± 3.30^{a}	23.70 ± 1.32^{b}	20.65 ± 1.00^{b}	10.50 ± 2.90^{a}	1.49 ± 0.004^{b}	0.69 ± 0.73^{b}

Table 2. Particulate organic (POC) and inorganic (PIC) carbon cell quotas for *E. huxleyi* (pg cell^{-1}) in HC-LP (1000-1200 µatm and pH 7.6-7.8), LC-HP (380-390 µatm and pH 8.1) and LC-LP (380-390 µatm and pH 7.6-7.8). Values are mean ± SD (n = 3). Significant differences between treatments are indicated by different letters for any given time ($p < 0.05$).

	***p*CO_2** (µatm)			**pH** (NBS Scale)			**$\Omega_{CALCITE}$**			**TA** (µmol kg SW^{-1})		
**Day**	**HC-LP**	**LC-HP**	**LC-LP**	**HC-LP**	**LC-HP**	**LC-LP**	**HC-LP**	**LC-HP**	**LC-LP**	**HC-LP**	**LC-HP**	**LC-LP**
0	1233.65 ± 59.00^{a}	456.75 ± 39.20^{b}	718.48 ± 76.87^{c}	7.62 ± 0.03^{b}	8.05 ± 0.05^{a}	7.60 ± 0.01^{b}	1.11 ± 0.06^{a}	2.54 ± 0.20^{b}	0.74 ± 0.03^{c}	1626.02 ± 56.33^{b}	1733.33 ± 153.12^{b}	991.87 ± 74.51^{a}
1	1221.82 ± 87.90^{a}	526.51 ± 50.20^{b}	628.26 ± 118.82^{b}	7.58 ± 0.06^{b}	7.94 ± 0.04^{a}	7.62 ± 0.07^{b}	0.90 ± 0.21^{b}	1.98 ± 0.13^{a}	0.52 ± 0.07^{b}	1593.50 ± 96.87^{b}	1678.05 ± 4.88^{b}	895.93 ± 22.00^{a}
2	1487.34 ± 98.65^{a}	570.54 ± 13.30^{b}	725.88 ± 92.14^{b}	7.47 ± 0.02^{b}	7.93 ± 0.02^{a}	7.49 ± 0.08^{b}	0.65 ± 0.02^{a}	2.04 ± 0.11^{b}	0.35 ± 0.09^{c}	1489.43 ± 57.17^{a}	1754.47 ± 25.03^{b}	767.48 ± 88.52^{c}
3	872.53 ± 47.54^{a}	397.60 ± 18.62^{b}	668.34 ± 183.07^{b}	7.70 ± 0.01^{b}	8.12 ± 0.01^{a}	7.55 ± 0.11^{b}	1.09 ± 0.03^{a}	3.50 ± 0.03^{b}	0.42 ± 0.09^{c}	1518.70 ± 53.73^{a}	2026.02 ± 45.06^{b}	808.13 ± 22.53^{c}
4	664.06 ± 64.41^{a}	294.14 ± 10.15^{b}	388.36 ± 51.10^{b}	7.79 ± 0.01^{b}	8.20 ± 0.01^{a}	7.76 ± 0.05^{b}	1.25 ± 0.14^{a}	3.66 ± 0.04^{b}	0.63 ± 0.05^{c}	1458.54 ± 180.69^{a}	1886.18 ± 14.90^{b}	800.00 ± 12.91^{c}
5	715.11 ± 24.33^{a}	218.60 ± 59.28^{b}	277.37 ± 102.92^{b}	7.76 ± 0.01^{b}	8.21 ± 0.08^{a}	7.73 ± 0.11^{b}	1.19 ± 0.13^{b}	2.93 ± 0.51^{a}	0.40 ± 0.05^{b}	1422.76 ± 77.49^{b}	1447.15 ± 161.59^{a}	521.95 ± 59.14^{b}
7	841.27 ± 82.73^{a}	189.50 ± 86.96^{b}	285.59 ± 42.56^{b}	7.64 ± 0.08^{b}	8.00 ± 0.14^{a}	7.68 ± 0.05^{b}	0.82 ± 0.21^{ab}	0.91 ± 0.30^{a}	0.33 ± 0.03^{b}	1245.53 ± 150.69^{a}	700.81 ± 144.24^{b}	487.80 ± 12.91^{b}
9	538.48 ± 143.68^{a}	188.75 ± 0.74^{b}	290.04 ± 3.71^{b}	7.58 ± 0.06^{b}	8.12 ± 0.01^{a}	7.62 ± 0.01^{b}	0.40 ± 0.004^{a}	0.83 ± 0.07^{b}	0.27 ± 0.01^{c}	669.92 ± 66.59^{a}	512.20 ± 27.16^{b}	416.26 ± 10.15^{b}

	**DIC** (µmol kg SW^{-1})			**CO_3^{2-}** (µmol kg SW^{-1})			**HCO_3^-** (µmol kg SW^{-1})			**CO_2** (µmol kg SW^{-1})		
**Day**	**HC-LP**	**LC-HP**	**LC-LP**	**HC-LP**	**LC-HP**	**LC-LP**	**HC-LP**	**LC-HP**	**LC-LP**	**HC-LP**	**LC-HP**	**LC-LP**
0	1576.96 ± 53.75^{b}	1558.32 ± 146.21^{b}	947.70 ± 76.87^{a}	49.93 ± 2.78^{a}	114.63 ± 9.19^{b}	30.91 ± 1.24^{c}	1492.23 ± 50.10^{b}	1430.21 ± 135.69^{b}	896.61 ± 73.42^{a}	34.80 ± 0.96^{a}	13.46 ± 1.34^{b}	20.17 ± 2.30^{c}
1	1571.37 ± 81.41^{b}	1557.04 ± 5.91^{b}	867.68 ± 28.39^{a}	38.05 ± 8.78^{b}	83.61 ± 5.61^{a}	22.12 ± 2.99^{b}	1491.36 ± 75.56^{b}	1455.75 ± 10.01^{b}	824.07 ± 27.11^{a}	41.96 ± 3.04^{a}	17.68 ± 1.51^{b}	21.49 ± 4.04^{b}
2	1492.73 ± 63.31^{b}	1633.64 ± 18.16^{b}	756.53 ± 82.06^{a}	27.27 ± 0.70^{a}	85.93 ± 4.69^{b}	14.64 ± 3.72^{c}	1414.75 ± 59.30^{b}	1528.70 ± 14.52^{b}	717.13 ± 79.29^{a}	50.71 ± 4.58^{a}	19.01 ± 0.61^{b}	24.75 ± 3.17^{b}
3	1469.15 ± 57.88^{a}	1811.71 ± 48.43^{b}	789.16 ± 23.35^{c}	45.78 ± 1.22^{a}	147.36 ± 1.15^{b}	17.56 ± 3.86^{c}	1393.91 ± 56.13^{a}	1651.30 ± 48.32^{b}	748.75 ± 21.97^{c}	29.46 ± 2.29^{ab}	13.04 ± 0.75^{b}	22.86 ± 6.09^{b}
4	1388.73 ± 177.52^{b}	1650.86 ± 16.94^{b}	748.47 ± 20.46^{a}	52.53 ± 6.15^{a}	154.30 ± 1.73^{b}	26.78 ± 2.09^{c}	1313.56 ± 168.55^{b}	1486.63 ± 17.46^{b}	708.60 ± 20.83^{a}	22.64 ± 2.81^{a}	9.93 ± 0.32^{b}	13.10 ± 1.72^{b}
5	1357.21 ± 70.64^{b}	1234.30 ± 161.29^{b}	476.91 ± 72.10^{a}	50.21 ± 5.40^{b}	123.60 ± 21.57^{a}	16.55 ± 2.11^{b}	1283.73 ± 65.07^{b}	1103.55 ± 156.36^{b}	451.24 ± 70.25^{a}	23.27 ± 0.31^{a}	7.15 ± 2.13^{b}	9.02 ± 3.35^{b}
7	1208.16 ± 136.32^{a}	604.07 ± 142.74^{b}	450.28 ± 16.34^{b}	34.62 ± 8.98^{ab}	38.38 ± 12.69^{a}	13.97 ± 1.42^{b}	1146.19 ± 128.65^{a}	559.50 ± 134.52^{b}	427.00 ± 15.87^{b}	27.35 ± 2.19^{a}	6.19 ± 2.90^{b}	9.32 ± 1.46^{b}
9	641.15 ± 73.34^{a}	391.80 ± 24.27^{b}	382.38 ± 9.43^{b}	16.98 ± 0.17^{b}	31.56 ± 6.56^{a}	11.43 ± 0.43^{b}	607.93 ± 69.37^{a}	357.54 ± 19.53^{b}	362.36 ± 8.89^{b}	16.24 ± 3.92^{a}	2.70 ± 0.06^{b}	8.60 ± 0.10^{b}

Table S1. Carbonate system. Measured pH, total alkalinity and calculated dissolved inorganic carbon (DIC), calcite saturation estate ($\Omega_{calcite}$), CO_3^{2-}, HCO_3^-, CO_2 and pCO_2 in the different treatments; HC-LP (pCO_2: 1000-1200 µatm; pH: 7.6-7.8), LC-HP (pCO_2: 380-390 µatm; pH: 8.2) and LC-LP (pCO_2: 380-390 µatm; pH: 7.6-7.8). Values are mean ± SD (n = 3). Significant differences between treatments are indicated by different letters for any given time ($p < 0.05$).

	L			*W*		
Day	HC-LP	LC-HP	LC-LP	HC-LP	LC-HP	LC-LP
0	6.39 ± 0.73[b]	7.32 ± 0.66[a]	6.06 ± 0.89[b]	5.98 ± 0.47[b]	7.04 ± 0.61[a]	5.72 ± 0.91[b]
1	7.63 ± 0.88[b]	7.28 ± 1.07[ab]	6.96 ± 0.68[a]	7.12 ± 0.78[b]	6.78 ± 0.87[ab]	6.50 ± 0.70[a]
2	7.47 ± 0.94[b]	6.58 ± 0.89[a]	7.08 ± 0.88[ab]	6.99 ± 0.95[b]	6.18 ± 0.76[a]	6.63 ± 0.69[ab]
4	8.22 ± 0.97[a]	6.94 ± 0.69[b]	7.33 ± 1.31[b]	7.71 ± 0.80[a]	6.52 ± 0.61[b]	6.82 ± 1.10[b]
7	5.78 ± 0.53[a]	5.29 ± 0.36[a]	5.65 ± 0.81[a]	5.26 ± 0.55[a]	4.99 ± 0.53[a]	5.08 ± 0.78[a]

Table S2. Variation of the morphology of the coccospheres in HC-LP, LC-HP, and LC-LP treatments. L, length of the coccosphere (µm); W, width of the coccosphere (µm). Values are mean ± SD (30 micrographs of coccoliths and coccospheres were analysed for every treatment and day). Significant differences between treatments are indicated by different letters for any given time ($p < 0.05$).

	DL			DW			CAL			CAW			$Seg.$		
**Day**	**HC-LP**	**LC-HP**	**LC-LP**	**HC-LP**	**LC-HP**	**LC-LP**	**HC-LP**	**LC-HP**	**LC-LP**	**HC-LP**	**LC-HP**	**LC-LP**	**HC-LP**	**LC-HP**	**LC-LP**
0	3.20 ± 0.25^{a}	3.69 ± 0.78^{a}	3.43 ± 0.28^{a}	2.67 ± 0.19^{a}	3.12 ± 0.59^{a}	2.87 ± 0.34^{a}	1.40 ± 0.08^{a}	1.66 ± 0.38^{a}	1.47 ± 0.15^{a}	0.75 ± 0.13^{a}	0.91 ± 0.19^{a}	0.88 ± 0.18^{a}	25.33 ± 4.27^{a}	26.33 ± 5.16^{a}	23.04 ± 4.32^{a}
1	3.44 ± 0.30^{b}	3.44 ± 0.26^{b}	3.18 ± 0.30^{a}	2.92 ± 0.34^{b}	2.92 ± 0.25^{b}	2.69 ± 0.30^{a}	1.50 ± 0.19^{a}	1.60 ± 0.19^{b}	1.47 ± 0.19^{b}	0.89 ± 0.20^{a}	0.95 ± 0.16^{a}	0.92 ± 0.16^{a}	25.57 ± 5.68^{a}	25.07 ± 4.21^{a}	24.63 ± 5.49^{a}
2	3.28 ± 0.35^{a}	3.27 ± 0.30^{a}	3.18 ± 0.32^{a}	2.79 ± 0.34^{a}	2.76 ± 0.28^{a}	2.65 ± 0.33^{a}	1.59 ± 0.25^{a}	1.63 ± 0.18^{a}	1.56 ± 0.22^{a}	0.94 ± 0.21^{a}	0.92 ± 0.15^{a}	0.94 ± 0.16^{a}	26.00 ± 4.74^{a}	24.96 ± 5.15^{a}	24.38 ± 3.11^{a}
4	3.33 ± 0.35^{a}	3.39 ± 0.24^{a}	3.27 ± 0.36^{a}	2.82 ± 0.37^{a}	2.87 ± 0.28^{a}	2.76 ± 0.36^{a}	1.58 ± 0.23^{a}	1.64 ± 0.18^{a}	1.56 ± 0.36^{a}	0.97 ± 0.19^{a}	0.95 ± 0.17^{a}	0.96 ± 0.17^{a}	23.69 ± 5.03^{a}	26.00 ± 4.61^{a}	25.64 ± 5.31^{a}
7	3.41 ± 0.29^{a}	3.16 ± 0.32^{b}	3.12 ± 0.28^{b}	2.88 ± 0.25^{a}	2.67 ± 0.31^{b}	2.63 ± 0.28^{b}	1.58 ± 0.18^{a}	1.54 ± 0.18^{a}	1.52 ± 0.20^{a}	0.98 ± 0.16^{a}	0.91 ± 0.19^{a}	0.88 ± 0.16^{a}	25.78 ± 4.84^{a}	23.64 ± 5.29^{a}	24.04 ± 5.02^{a}

	DSA			CSA			OSL			$TW_{relative}$			m		
**Day**	**HC-LP**	**LC-HP**	**LC-LP**	**HC-LP**	**LC-HP**	**LC-LP**	**HC-LP**	**LC-HP**	**LC-LP**	**HC-LP**	**LC-HP**	**LC-LP**	**HC-LP**	**LC-HP**	**LC-LP**
0	6.73 ± 0.99^{a}	9.35 ± 4.25^{a}	7.79 ± 1.51^{a}	0.83 ± 0.17^{a}	1.23 ± 0.56^{a}	1.02 ± 0.27^{a}	0.93 ± 0.10^{a}	1.06 ± 0.22^{a}	0.99 ± 0.10^{a}	0.20 ± 0.03^{b}	0.24 ± 0.02^{a}	0.20 ± 0.03^{b}	2.69 ± 0.65^{a}	3.43 ± 0.96^{a}	3.31 ± 0.81^{a}
1	7.90 ± 1.58^{b}	7.93 ± 1.23^{b}	6.77 ± 1.41^{a}	1.07 ± 0.38^{a}	1.21 ± 0.33^{a}	1.07 ± 0.30^{a}	0.99 ± 0.11^{b}	0.95 ± 0.11^{b}	0.87 ± 0.12^{a}	0.24 ± 0.04^{b}	0.23 ± 0.04^{ab}	0.21 ± 0.03^{a}	3.37 ± 0.90^{b}	3.34 ± 0.76^{b}	2.67 ± 0.78^{a}
2	7.27 ± 1.61^{a}	7.14 ± 1.36^{a}	6.70 ± 1.51^{a}	1.21 ± 0.44^{a}	1.18 ± 0.30^{a}	1.17 ± 0.33^{a}	0.88 ± 0.10^{a}	0.87 ± 0.10^{a}	0.83 ± 0.11^{a}	0.24 ± 0.04^{b}	0.24 ± 0.04^{b}	0.21 ± 0.03^{a}	2.95 ± 0.93^{a}	2.90 ± 0.79^{a}	2.68 ± 0.83^{a}
4	7.48 ± 1.82^{a}	7.72 ± 1.48^{a}	7.17 ± 1.72^{a}	1.22 ± 0.40^{a}	1.23 ± 0.33^{a}	1.19 ± 0.34^{a}	0.90 ± 0.13^{a}	0.92 ± 0.13^{a}	0.88 ± 0.16^{a}	0.20 ± 0.04^{b}	0.26 ± 0.09^{a}	0.19 ± 0.04^{b}	2.98 ± 0.70^{a}	3.23 ± 0.86^{a}	2.95 ± 0.88^{a}
7	7.77 ± 1.32^{a}	6.71 ± 1.47^{b}	6.49 ± 1.26^{b}	1.23 ± 0.32^{a}	1.12 ± 0.34^{a}	1.07 ± 0.31^{a}	0.93 ± 0.11^{a}	0.85 ± 0.11^{b}	0.84 ± 0.11^{b}	0.21 ± 0.04^{a}	0.22 ± 0.07^{a}	0.21 ± 0.03^{a}	3.28 ± 0.83^{b}	2.59 ± 0.62^{ab}	2.51 ± 0.68^{a}

	DR			CAR		
**Day**	**HC-LP**	**LC-HP**	**LC-LP**	**HC-LP**	**LC-HP**	**LC-LP**
0	83.56 ± 2.67^{a}	84.93 ± 3.27^{a}	83.69 ± 4.43^{a}	53.74 ± 8.09^{a}	55.19 ± 7.06^{a}	59.95 ± 9.81^{a}
1	84.75 ± 3.71^{a}	85.08 ± 3.30^{a}	84.32 ± 2.94^{a}	58.87 ± 8.97^{a}	59.60 ± 6.94^{a}	62.41 ± 7.46^{a}
2	84.89 ± 3.74^{a}	84.42 ± 3.57^{a}	83.17 ± 3.59^{a}	58.95 ± 7.27^{ab}	56.36 ± 6.56^{a}	60.48 ± 6.22^{b}
4	84.60 ± 3.55^{a}	84.86 ± 3.50^{a}	84.49 ± 4.57^{a}	61.24 ± 7.48^{a}	57.86 ± 8.99^{a}	61.65 ± 8.42^{a}
7	84.54 ± 2.12^{a}	84.42 ± 3.08^{a}	84.21 ± 3.12^{a}	62.47 ± 7.35^{b}	58.75 ± 7.64^{ab}	57.89 ± 7.46^{a}

Table S3. Variation of the morphology of the coccoliths in HC-LP, LC-HP, and LC-LP treatments. DL, length of the distal plate (µm): DW, width of the distal plate (µm); CAL, center area length (µm); CAW, width of the central area (µm); Seg., number of segments; DSA, surface area of the distal shield ($µm^2$); CSA, surface area of the central shield ($µm^2$); OSL, distal shield width (µm); TWrelative, relative width of the tube; m, coccolith mass (pg), DR, roundness of the distal shield (%); CAR, roundness of the central area (%). Values are mean ± SD (30 micrographs of coccoliths and coccospheres were analysed for every treatment and day). Significant differences between treatments are indicated by different letters for any given time ($p < 0.05$).

Table S4. Statistical analyses (Split-Plot ANOVA followed by post-hoc Sidak and Bonferroni tests) of the effects of treatments, as well as the effect of time, on the variables analysed in the different treatments during the experiment. Statistically significant differences ($p < 0.05$) are indicated with an asterisk (*); ns: not significant ($p > 0.05$).

Factor/variable	Treatment	Time	Treatment x Time
Cellular density (cells · ml^{-1})	< 0.001*	< 0.001*	< 0.001*
Chl a (pg · $cells^{-1}$)	< 0.001*	< 0.001*	< 0.001*
F_V/F_M	< 0.001*	< 0.001*	< 0.001*
Cell viability (%)	< 0.001*	< 0.001*	< 0.001*
Oxidative stress (%)	< 0.001*	< 0.001*	< 0.001*
TPC (µmol · L^{-1})	< 0.001*	< 0.001*	< 0.001*
POC (µmol · L^{-1})	< 0.001*	< 0.001*	< 0.001*
PIC (µmol · L^{-1})	= 0.003*	< 0.001*	= 0.007*
PIC:POC ratio (mol: mol)	= 0.001*	ns	= 0.002*
α_{ETR} (e^- · $photons^{-1}$)	= 0.001*	< 0.001*	< 0.001*
ETR_{max} (µmol e^- · m^{-2} · s^{-1})	= 0.024*	< 0.001*	< 0.001*
E_k (µmol photons · m^{-2} · s^{-1})	ns	< 0.001*	< 0.001*
E_{opt} ETR (µmol photons · m^{-2} · s^{-1})	ns	ns	ns
pH (NBS Scale)	< 0.001*	< 0.001*	ns
TA (µmol · kg · SW^{-1})	< 0.001*	< 0.001*	< 0.001*
DIC (µmol kg · SW^{-1})	< 0.001*	< 0.001*	< 0.001*
$\Omega_{calcite}$	< 0.001*	< 0.001*	< 0.001*
CO_3^{2-} (µmol kg · SW^{-1})	< 0.001*	< 0.001*	< 0.001*
HCO_3^- (µmol kg · SW^{-1})	< 0.001*	< 0.001*	< 0.001*
CO_2 (µmol kg · SW^{-1})	< 0.001*	< 0.001*	= 0.026*
pCO_2 (µatm)	< 0.001*	< 0.001*	= 0.039*
L (µm)	ns	ns	= 0.003*
W (µm)	ns	< 0.001*	= 0.049*
DL (µm)	= 0.050*	= 0.049*	= 0.032*
DW (µm)	ns	= 0.050*	= 0.013*
CAL (µm)	ns	ns	= 0.002*
CAW (µm)	ns	ns	= 0.003*
Seg. (number of segments)	ns	= 0.037*	ns
DSA ($µm^2$)	ns	= 0.043*	= 0.022*
CSA ($µm^2$)	ns	ns	= 0.002*
OSL (µm)	ns	= 0.002*	ns
$TW_{relative}$	< 0.001*	ns	ns
m (pg)	ns	ns	ns
DR (%)	ns	ns	ns
CAR (%)	ns	ns	= 0.013*
POC (pg $cell^{-1}$)	< 0.001*	< 0.001*	< 0.001*
PIC (pg $cell^{-1}$)	< 0.001*	< 0.001*	< 0.001*

Factor/variable	HC-LP vs. LC-LP	HC-LP vs. LC-HP	LC-LP vs. LC-HP
Cellular density (cells · ml^{-1})	< 0.001*	< 0.001*	< 0.001*
Chl a (pg · $cells^{-1}$)	= 0.003*	< 0.001*	= 0.001*
F_V/F_M	< 0.001*	< 0.001*	ns
Cell viability (%)	< 0.001*	< 0.001*	< 0.001*
Oxidative stress (%)	< 0.001*	< 0.001*	ns
TPC (µmol · L^{-1})	= 0.004*	< 0.001*	= 0.007*
POC (µmol · L^{-1})	< 0.001*	< 0.001*	< 0.001*
PIC (µmol · L^{-1})	ns	= 0.003*	= 0.039*
PIC:POC ratio (mol: mol)	= 0.011*	= 0.001*	ns
α_{ETR} (e^- · $photons^{-1}$)	= 0.006*	= 0.003*	ns
ETR_{max} (µmol e^- · m^{-2} · s^{-1})	ns	= 0.027*	ns
E_k (µmol photons · m^{-2} · s^{-1})	ns	ns	ns
E_{opt} ETR (µmol photons · m^{-2} · s^{-1})	ns	ns	ns
pH (NBS Scale)	ns	< 0.001*	< 0.001*
TA (µmol · kg · SW^{-1})	< 0.001*	ns	< 0.001*
DIC (µmol kg · SW^{-1})	< 0.001*	ns	< 0.001*
$\Omega_{calcite}$	= 0.003*	< 0.001*	< 0.001*
CO_3^{2-} (µmol kg · SW^{-1})	= 0.004*	< 0.001*	< 0.001*
HCO_3^- (µmol kg · SW^{-1})	< 0.001*	ns	< 0.001*
CO_2 (µmol kg · SW^{-1})	< 0.001*	< 0.001*	= 0.006*
pCO_2 (µatm)	< 0.001*	< 0.001*	= 0.003*
L (µm)	ns	ns	ns
W (µm)	ns	ns	ns
DL (µm)	ns	ns	ns
DW (µm)	ns	ns	ns
CAL (µm)	ns	ns	ns
CAW (µm)	ns	ns	ns
Seg. (number of segments)	ns	ns	ns
DSA ($µm^2$)	ns	ns	ns
CSA ($µm^2$)	ns	ns	ns
OSL (µm)	ns	ns	ns
$TW_{relative}$	ns	= 0.008*	< 0.001*
m (pg)	ns	ns	ns
DR (%)	ns	ns	ns
CAR (%)	ns	ns	ns
POC (pg $cell^{-1}$)	< 0.001*	< 0.001*	ns
PIC (pg $cell^{-1}$)	< 0.001*	< 0.001*	ns

		Cellular density (cells · ml^{-1})	Chl a (pg · cells^{-1})	F_V/F_M	Cell viability (%)	Oxidative stress (%)
d_0 (0 h)	HC-LP vs. LC-LP	-	-	< 0.001*	< 0.001*	ns
	HC-LP vs. LC-HP	-	-	< 0.001*	< 0.001*	ns
	LC-LP vs. LC-HP	-	-	= 0.003*	< 0.001*	ns
d_1 (24 h)	HC-LP vs. LC-LP	< 0.001*	-	-	-	-
	HC-LP vs. LC-HP	< 0.001*	-	-	-	-
	LC-LP vs. LC-HP	= 0.002*	-	-	-	-
d_2 (48 h)	HC-LP vs. LC-LP	< 0.001*	ns	< 0.001*	< 0.001*	ns
	HC-LP vs. LC-HP	< 0.001*	= 0.006*	< 0.001*	ns	= 0.001*
	LC-LP vs. LC-HP	= 0.621	ns	ns	< 0.001*	< 0.001*
d_3 (72 h)	HC-LP vs. LC-LP	< 0.001*	-	-	-	-
	HC-LP vs. LC-HP	< 0.001*	-	-	-	-
	LC-LP vs. LC-HP	= 0.401	-	-	-	-
d_4 (96 h)	HC-LP vs. LC-LP	< 0.001*	= 0.011*	= 0.042*	< 0.001*	< 0.001*
	HC-LP vs. LC-HP	< 0.001*	= 0.002*	ns	< 0.001*	< 0.001*
	LC-LP vs. LC-HP	< 0.001*	ns	ns	< 0.001*	= 0.001*
d_5 (120 h)	HC-LP vs. LC-LP	< 0.001*	-	-	-	-
	HC-LP vs. LC-HP	< 0.001*	-	-	-	-
	LC-LP vs. LC-HP	< 0.001*	-	-	-	-
d_7 (168 h)	HC-LP vs. LC-LP	< 0.001*	= 0.033*	= 0.002*	< 0.001*	< 0.001*
	HC-LP vs. LC-HP	< 0.001*	= 0.002*	= 0.020*	< 0.001*	< 0.001*
	LC-LP vs. LC-HP	< 0.001*	ns	ns	< 0.001*	< 0.001*
d_8 (192 h)	HC-LP vs. LC-LP	< 0.001*	-	-	-	-
	HC-LP vs. LC-HP	< 0.001*	-	-	-	-
	LC-LP vs. LC-HP	= 0.002*	-	-	-	-
d_9 (216 h)	HC-LP vs. LC-LP	< 0.001*	= 0.010*	= 0.017*	< 0.001*	< 0.001*
	HC-LP vs. LC-HP	< 0.001*	< 0.001*	ns	< 0.001*	< 0.001*
	LC-LP vs. LC-HP	< 0.001*	= 0.001*	ns	< 0.001*	= 0.001*

		TPC (µmol · L^{-1})	POC (µmol · L^{-1})	PIC (µmol · L^{-1})	PIC:POC ratio (mol: mol)	α_{ETR} (e$^-$ · photons^{-1})	$rETR_{max}$ (µmol e$^-$ · m^{-2} · s^{-1})
d_0 (0 h)	HC-LP vs. LC-LP	< 0.001*	ns	< 0.001*	< 0.001*	< 0.001*	= 0.001*
	HC-LP vs. LC-HP	ns	ns	= 0.007*	< 0.001*	< 0.001*	< 0.001*
	LC-LP vs. LC-HP	< 0.001*	ns	< 0.001*	= 0.001*	ns	ns
d_1 (24 h)	HC-LP vs. LC-LP	-	-	-	-	-	-
	HC-LP vs. LC-HP	-	-	-	-	-	-
	LC-LP vs. LC-HP	-	-	-	-	-	-
d_2 (48 h)	HC-LP vs. LC-LP	ns	ns	ns	ns	= 0.012*	= 0.004*
	HC-LP vs. LC-HP	ns	ns	ns	ns	= 0.036*	ns
	LC-LP vs. LC-HP	ns	ns	ns	ns	ns	ns
d_3 (72 h)	HC-LP vs. LC-LP	-	-	-	-	-	-
	HC-LP vs. LC-HP	-	-	-	-	-	-
	LC-LP vs. LC-HP	-	-	-	-	-	-
d_4 (96 h)	HC-LP vs. LC-LP	= 0.004*	< 0.001*	ns	ns	ns	ns
	HC-LP vs. LC-HP	ns	< 0.001*	= 0.007*	= 0.002*	= 0.022*	= 0.037*
	LC-LP vs. LC-HP	= 0.048*	= 0.004*	= 0.002*	= 0.014*	ns	ns
d_5 (120 h)	HC-LP vs. LC-LP	-	-	-	-	-	-
	HC-LP vs. LC-HP	-	-	-	-	-	-
	LC-LP vs. LC-HP	-	-	-	-	-	-
d_7 (168 h)	HC-LP vs. LC-LP	= 0.037*	= 0.008*	ns	ns	ns	< 0.001*
	HC-LP vs. LC-HP	= 0.011*	< 0.001*	= 0.015*	ns	ns	= 0.006*
	LC-LP vs. LC-HP	ns	= 0.007*	= 0.003*	ns	ns	ns
d_8 (192 h)	HC-LP vs. LC-LP	-	-	-	-	-	-
	HC-LP vs. LC-HP	-	-	-	-	-	-
	LC-LP vs. LC-HP	-	-	-	-	-	-
d_9 (216 h)	HC-LP vs. LC-LP	= 0.006*	< 0.001*	= 0.015*	= 0.002*	ns	ns
	HC-LP vs. LC-HP	< 0.001*	< 0.001*	ns	= 0.002*	ns	ns
	LC-LP vs. LC-HP	< 0.001*	< 0.001*	ns	ns	ns	ns

		E_k (µmol photons · m^{-2} · s^{-1})	E_{opt} ETR (µmol photons · m^{-2} · s^{-1})	pH (NBS Scale)	TA (µmol · kg · SW^{-1})	DIC (µmol kg · SW^{-1})	$\Omega_{calcite}$
d_0 (0 h)	HC-LP vs. LC-LP	ns	ns	ns	= 0.001*	= 0.001*	= 0.031*
	HC-LP vs. LC-HP	ns	ns	< 0.001*	ns	ns	< 0.001*
	LC-LP vs. LC-HP	= 0.007*	ns	< 0.001*	< 0.001*	= 0.001*	< 0.001*
d_1 (24 h)	HC-LP vs. LC-LP	-	-	ns	< 0.001*	< 0.001*	ns
	HC-LP vs. LC-HP	-	-	= 0.001*	ns	ns	< 0.001*
	LC-LP vs. LC-HP	-	-	= 0.001*	< 0.001*	< 0.001*	< 0.001*
d_2 (48 h)	HC-LP vs. LC-LP	= 0.037*	ns	ns	< 0.001*	< 0.001*	= 0.013*
	HC-LP vs. LC-HP	ns	ns	< 0.001*	= 0.006*	ns	< 0.001*
	LC-LP vs. LC-HP	ns	ns	< 0.001*	< 0.001*	< 0.001*	< 0.001*
d_3 (72 h)	HC-LP vs. LC-LP	-	-	ns	< 0.001*	< 0.001*	< 0.001*
	HC-LP vs. LC-HP	-	-	= 0.001*	< 0.001*	< 0.001*	< 0.001*
	LC-LP vs. LC-HP	-	-	< 0.001*	< 0.001*	< 0.001*	< 0.001*
d_4 (96 h)	HC-LP vs. LC-LP	ns	ns	ns	= 0.001*	= 0.001*	= 0.001*
	HC-LP vs. LC-HP	ns	ns	< 0.001*	= 0.007*	ns	< 0.001*
	LC-LP vs. LC-HP	ns	ns	< 0.001*	< 0.001*	< 0.001*	< 0.001*
d_5 (120 h)	HC-LP vs. LC-LP	-	-	ns	< 0.001*	< 0.001*	ns
	HC-LP vs. LC-HP	-	-	= 0.001*	ns	ns	= 0.001*
	LC-LP vs. LC-HP	-	-	= 0.001*	< 0.001*	< 0.001*	< 0.001*
d_7 (168 h)	HC-LP vs. LC-LP	= 0.016*	= 0.001*	ns	= 0.001*	= 0.001*	ns
	HC-LP vs. LC-HP	= 0.043*	= 0.011*	< 0.001*	= 0.004*	= 0.002*	ns
	LC-LP vs. LC-HP	ns	ns	< 0.001*	ns	ns	= 0.048*
d_8 (192 h)	HC-LP vs. LC-LP	-	-	-	-	-	-
	HC-LP vs. LC-HP	-	-	-	-	-	-
	LC-LP vs. LC-HP	-	-	-	-	-	-
d_9 (216 h)	HC-LP vs. LC-LP	= 0.015*	ns	ns	= 0.001*	= 0.001*	= 0.019*
	HC-LP vs. LC-HP	= 0.030*	ns	< 0.001*	= 0.011*	= 0.001*	< 0.001*
	LC-LP vs. LC-HP	ns	ns	< 0.001*	ns	ns	< 0.001*

		CO_3^{2-} (µmol kg · SW^{-1})	HCO_3^- (µmol kg · SW^{-1})	CO_2 (µmol kg · SW^{-1})	pCO_2 (µatm)	L (µm)	W (µm)
d_0 (0 h)	HC-LP vs. LC-LP	= 0.018*	= 0.001*	< 0.001*	< 0.001*	ns	ns
	HC-LP vs. LC-HP	< 0.001*	ns	< 0.001*	< 0.001*	= 0.034*	= 0.005*
	LC-LP vs. LC-HP	< 0.001*	= 0.001*	= 0.007*	= 0.004*	= 0.004*	= 0.001*
d_1 (24 h)	HC-LP vs. LC-LP	ns	< 0.001*	= 0.001*	= 0.001*	= 0.013*	= 0.008*
	HC-LP vs. LC-HP	< 0.001*	ns	< 0.001*	< 0.001*	ns	ns
	LC-LP vs. LC-HP	< 0.001*	< 0.001*	ns	ns	ns	ns
d_2 (48 h)	HC-LP vs. LC-LP	= 0.013*	< 0.001*	< 0.001*	< 0.001*	ns	ns
	HC-LP vs. LC-HP	< 0.001*	ns	< 0.001*	< 0.001*	= 0.001*	= 0.001*
	LC-LP vs. LC-HP	< 0.001*	< 0.001*	ns	ns	ns	ns
d_3 (72 h)	HC-LP vs. LC-LP	< 0.001*	< 0.001*	ns	ns	-	-
	HC-LP vs. LC-HP	< 0.001*	= 0.001*	= 0.005*	= 0.005*	-	-
	LC-LP vs. LC-HP	< 0.001*	< 0.001*	ns	ns	-	-
d_4 (96 h)	HC-LP vs. LC-LP	= 0.001*	= 0.001*	= 0.003*	= 0.001*	= 0.005*	= 0.001*
	HC-LP vs. LC-HP	< 0.001*	ns	= 0.001*	< 0.001*	< 0.001*	< 0.001*
	LC-LP vs. LC-HP	< 0.001*	< 0.001*	ns	ns	ns	ns
d_5 (120 h)	HC-LP vs. LC-LP	ns	< 0.001*	= 0.001*	= 0.001*	-	-
	HC-LP vs. LC-HP	= 0.001*	ns	< 0.001*	< 0.001*	-	-
	LC-LP vs. LC-HP	< 0.001*	= 0.001*	ns	ns	-	-
d_7 (168 h)	HC-LP vs. LC-LP	ns	= 0.001*	< 0.001*	< 0.001*	ns	ns
	HC-LP vs. LC-HP	ns	= 0.002*	< 0.001*	< 0.001*	ns	ns
	LC-LP vs. LC-HP	= 0.047*	ns	ns	ns	ns	ns
d_8 (192 h)	HC-LP vs. LC-LP	-	-	-	-	-	-
	HC-LP vs. LC-HP	-	-	-	-	-	-
	LC-LP vs. LC-HP	-	-	-	-	-	-
d_9 (216 h)	HC-LP vs. LC-LP	ns	= 0.001*	= 0.018*	= 0.031*	-	-
	HC-LP vs. LC-HP	= 0.010*	= 0.001*	= 0.001*	= 0.002*	-	-
	LC-LP vs. LC-HP	= 0.002*	ns	ns	ns	-	-

		DL (µm)	DW (µm)	CAL (µm)	CAW (µm)	Seg. (number of segments)	DSA (µm^2)	CSA (µm^2)
d_0 (0 h)	HC-LP vs. LC-LP	ns	ns	ns	ns	ns	ns	ns
	HC-LP vs. LC-HP	ns	ns	ns	ns	ns	ns	ns
	LC-LP vs. LC-HP	ns	ns	ns	ns	ns	ns	ns
d_1 (24 h)	HC-LP vs. LC-LP	= 0.002*	= 0.009*	ns	ns	ns	= 0.005*	ns
	HC-LP vs. LC-HP	ns	ns	ns	ns	ns	ns	ns
	LC-LP vs. LC-HP	= 0.003*	= 0.008*	= 0.028*	ns	ns	= 0.007*	ns
d_2 (48 h)	HC-LP vs. LC-LP	ns	ns	ns	ns	ns	ns	ns
	HC-LP vs. LC-HP	ns	ns	ns	ns	ns	ns	ns
	LC-LP vs. LC-HP	ns	ns	ns	ns	ns	ns	ns
d_3 (72 h)	HC-LP vs. LC-LP	-	-	-	-	-	-	-
	HC-LP vs. LC-HP	-	-	-	-	-	-	-
	LC-LP vs. LC-HP	-	-	-	-	-	-	-
d_4 (96 h)	HC-LP vs. LC-LP	ns	ns	ns	ns	ns	ns	ns
	HC-LP vs. LC-HP	ns	ns	ns	ns	ns	ns	ns
	LC-LP vs. LC-HP	ns	ns	ns	ns	ns	ns	ns
d_5 (120 h)	HC-LP vs. LC-LP	-	-	-	-	-	-	-
	HC-LP vs. LC-HP	-	-	-	-	-	-	-
	LC-LP vs. LC-HP	-	-	-	-	-	-	-
d_7 (168 h)	HC-LP vs. LC-LP	= 0.001*	= 0.002*	ns	ns	ns	= 0.001*	ns
	HC-LP vs. LC-HP	= 0.007*	= 0.016*	ns	ns	ns	= 0.010*	ns
	LC-LP vs. LC-HP	ns	ns	ns	ns	ns	ns	ns
d_8 (192 h)	HC-LP vs. LC-LP	-	-	-	-	-	-	-
	HC-LP vs. LC-HP	-	-	-	-	-	-	-
	LC-LP vs. LC-HP	-	-	-	-	-	-	-
d_9 (216 h)	HC-LP vs. LC-LP	-	-	-	-	-	-	-
	HC-LP vs. LC-HP	-	-	-	-	-	-	-
	LC-LP vs. LC-HP	-	-	-	-	-	-	-

		OSL (µm)	$TW_{relative}$	m (pg)	DR (%)	CAR (%)	POC (pg cell^{-1})	PIC (pg cell^{-1})
d_0 (0 h)	HC-LP vs. LC-LP	ns	ns	ns	ns	ns	ns	< 0.001*
	HC-LP vs. LC-HP	ns	= 0.024*	ns	ns	ns	ns	= 0.011*
	LC-LP vs. LC-HP	ns	= 0.017*	ns	ns	ns	ns	< 0.001*
d_1 (24 h)	HC-LP vs. LC-LP	< 0.001*	= 0.016*	= 0.004*	ns	ns	-	-
	HC-LP vs. LC-HP	ns	ns	ns	ns	ns	-	-
	LC-LP vs. LC-HP	= 0.025*	ns	= 0.007*	ns	ns	-	-
d_2 (48 h)	HC-LP vs. LC-LP	ns	= 0.003*	ns	ns	ns	< 0.001*	= 0.001*
	HC-LP vs. LC-HP	ns	ns	ns	ns	ns	< 0.001*	= 0.001*
	LC-LP vs. LC-HP	ns	= 0.002*	ns	ns	= 0.042*	ns	ns
d_3 (72 h)	HC-LP vs. LC-LP	-	-	-	-	-	-	-
	HC-LP vs. LC-HP	-	-	-	-	-	-	-
	LC-LP vs. LC-HP	-	-	-	-	-	-	-
d_4 (96 h)	HC-LP vs. LC-LP	ns	ns	ns	ns	ns	< 0.001*	= 0.001*
	HC-LP vs. LC-HP	ns	= 0.001*	ns	ns	ns	< 0.001*	= 0.001*
	LC-LP vs. LC-HP	ns	< 0.001*	ns	ns	ns	ns	ns
d_5 (120 h)	HC-LP vs. LC-LP	-	-	-	-	-	-	-
	HC-LP vs. LC-HP	-	-	-	-	-	-	-
	LC-LP vs. LC-HP	-	-	-	-	-	-	-
d_7 (168 h)	HC-LP vs. LC-LP	= 0.003*	ns	< 0.001*	ns	= 0.042*	= 0.002*	= 0.001*
	HC-LP vs. LC-HP	= 0.009*	ns	ns	ns	ns	= 0.007*	< 0.001*
	LC-LP vs. LC-HP	ns	ns	ns	ns	ns	ns	ns
d_8 (192 h)	HC-LP vs. LC-LP	-	-	-	-	-	-	-
	HC-LP vs. LC-HP	-	-	-	-	-	-	-
	LC-LP vs. LC-HP	-	-	-	-	-	-	-
d_9 (216 h)	HC-LP vs. LC-LP	-	-	-	-	-	< 0.001*	= 0.001*
	HC-LP vs. LC-HP	-	-	-	-	-	= 0.001*	= 0.002*
	LC-LP vs. LC-HP	-	-	-	-	-	ns	ns

Factor/variable	Treatment
K_M (μM)	= 0.002*
V_{max} (nmol C · 10^6 cells^{-1} · h^{-1})	ns
δ^{13} $C_{microalgae}$ (‰)	< 0.001*
pH compensation point	< 0.001*

Factor/variable	HC-LP vs. LC-LP	HC-LP vs. LC-HP	LC-LP vs. LC-HP
K_M (μM)	ns	= 0.004*	= 0.004*
V_{max} (nmol C · 10^6 cells^{-1} · h^{-1})	ns	ns	ns
δ^{13} $C_{microalgae}$ (‰)	< 0.001*	< 0.001*	ns
pH compensation point	< 0.001*	< 0.001*	< 0.001*

Table S5. Statistical significance of the effects of the treatments (CO_2 and pH) on the C fixation photosynthetic parameters analyzed by one-way ANOVAs. Significant differences ($p < 0.05$) are indicated with an asterisk (*); ns: not significant ($p > 0.05$).
