# Supplementary material for: High-CO2 Levels Rather than Acidification Restrict Emiliania huxleyi Growth and Performance
Source: Microb Ecol. 2022 May 27;86(1):127–43. doi: 10.1007/s00248-022-02035-3 (PMC10293452; doi:10.1007/s00248-022-02035-3)
Supplement: Supplementary file 2 — Supplementary file2 (PDF 407 KB) [file 248_2022_2035_MOESM2_ESM.pdf]

Adis · Bohn Stafleu van Loghum · palgrave macmillan · Springer

Change of authorship request form (pre-acceptance)

Please read the important information on page 4 before you begin

This form should be used by authors to request any change in authorship including changes in corresponding authors. Please fully complete all sections. Use black ink and block capitals and provide each author's full name with the given name first followed by the family name.

Please note: In author collaborations where there is formal agreement for representing the collaboration, it is sufficient for the representative or legal guarantor (usually the corresponding author) to complete and sign the Authorship Change Form on behalf of all authors.

**Manuscript ID no. in case of unpublished manuscript:** MECO-D-21-00562	
**DOI in case of published manuscript:**	
**ISBN (for books):**	

Section 1: Please provide the current title of manuscript
(For journals: Please provide the manuscript ID, title and/or DOI if available.)
(For books: Please provide the title, ISBN and/or DOI if available.)

Title: **High CO_2 levels rather than acidification restrict *Emiliania huxleyi* growth and performance**

Section 2: Please provide the previous authorship, in the order shown on the manuscript before the changes were introduced. Please indicate the corresponding author by adding (CA) behind the name.

	First name(s)	Family name	ORCID or SCOPUS id, if available
1st author	Víctor (CA)	Vazquez	https://orcid.org/0000-0002-0695-4150
2nd author	Pablo	León	https://orcid.org/0000-0002-7018-4381
3rd author	Francisco J.	L. Gordillo	https://orcid.org/0000-0003-1302-7213
4th author	Carlos	Jiménez	https://orcid.org/0000-0003-1025-8059
5th author	Kevin	Mackenzie	https://orcid.org/0000-0002-0041-5375
6th author	Eileen	Bresnan	https://orcid.org/0000-0002-4844-3295
7th author	María	Segovia	https://orcid.org/0000-0002-9765-5817

Please use an additional sheet if there are more than 7 authors.

SPRINGER NATURE Springer Nature is one of the world's leading global research, educational and professional publishers, created in May 2015 through the combination of Nature Publishing Group, Palgrave Macmillan, Macmillan Education and Springer Science+Business Media.

Change of authorship request form (pre-acceptance)

Section 3: Please provide a justification for change. Please use this section to explain your reasons for changing the authorship of your manuscript, e.g. what necessitated the change in authorship? Please refer to the (journal) policy pages for more information about authorship. Please explain why omitted authors were not originally included and/or why authors were removed on the submitted manuscript.

Concepcion Iñiguez (5th author) measured the photosynthesis curves with ^{14}C and has been key on revising the manuscript and answering some of the questions rised by the reviewers.

Section 4: Proposed new authorship. Please provide your new authorship list in the order you would like it to appear on the manuscript. Please indicate the corresponding author by adding (CA) behind the name. <u>If the corresponding author has changed, please indicate the reason under section 3.</u>

	First name(s)	Family name (this name will appear in full on the final publication and will be searchable in various abstract and indexing databases)
1st author	Víctor (CA)	Vazquez
2nd author	Pablo	León
3rd author	Francisco J.	L. Gordillo
4th author	Carlos	Jiménez
5th author	Concepción	Iñiguez
6th author	Kevin	Mackenzie
7th author	Eileen	Bresnan
8th author	María	Segovia

Please use an additional sheet if there are more than 7 authors.

SPRINGER NATURE Springer Nature is one of the world's leading global research, educational and professional publishers, created in May 2015 through the combination of Nature Publishing Group, Palgrave Macmillan, Macmillan Education and Springer Science+Business Media.

Change of authorship request form (pre-acceptance)

Section 5: Author contribution, Acknowledgement and Disclosures. Please use this section to provide a new disclosure statement and, if appropriate, acknowledge any contributors who have been removed as authors and ensure you state what contribution any new authors made (if applicable per the journal or book (series) policy). Please ensure these are updated in your manuscript - after approval of the change(s) - as our production department will not transfer the information in this form to your manuscript.

New acknowledgements:

New Disclosures (financial and non-financial interests, funding):

New Author Contributions statement (if applicable per the journal policy):

V.V.: Run the experiments, worked in the lab, analysed samples and data, wrote the paper.

P.L.: Analysed samples and data, wrote the paper.

F.G.: Designed the experiment, worked in the lab, discussed results, wrote the paper.

C.J.: Designed the experiment, worked in the lab, discussed results, wrote the paper.

C.I.: Analysed samples.

K.M.: Analysed samples.

E.B.: Analysed samples.

M.S.: Designed the experiment, discussed results, wrote the paper.

State 'Not applicable' if there are no new authors.

Change of authorship request form (pre-acceptance)

Section 6: Declaration of agreement. *All* authors, unchanged, new and removed *must* sign this declaration.
(NB: Please print the form, sign and return a scanned copy. Please note that signatures that have been inserted as an image file are acceptable as long as it is handwritten. Typed names in the signature box are unacceptable.) *** Please delete as appropriate. Delete all of the bold if you were on the original authorship list and are remaining as an author.**

	First name	Family name		Signature	Affiliated institute	Date
1st author	Víctor	Vázquez	I agree to the proposed new authorship shown in section 4		Department of Ecology, Faculty of Sciences, University of Málaga, Boulevard Louis Pasteur s/n, 29071-Málaga, Spain.	02.12.2021
2nd author	Pablo	León	I agree to the proposed new authorship shown in section 4		Marine Scotland Science, Marine Laboratory, 375 Victoria Road, Aberdeen, AB11 9DB, UK.	02/12/2021
3rd author	Francisco J.	L. Gordillo	I agree to the proposed new authorship shown in section 4		Department of Ecology, Faculty of Sciences, University of Málaga, Boulevard Louis Pasteur s/n, 29071-Málaga, Spain.	02/12/2021
4th author	Carlos	Jiménez	I agree to the proposed new authorship shown in section 4		Department of Ecology, Faculty of Sciences, University of Málaga, Boulevard Louis Pasteur s/n, 29071-Málaga, Spain.	02/12/2021
5th author	Concepción	Iñiguez	I agree to the proposed new authorship shown in section 4 **and the addition of my name to the authorship list.**		Department of Ecology, Faculty of Sciences, University of Málaga, Boulevard Louis Pasteur s/n, 29071-Málaga, Spain.	02/12/2021
6th author	Kevin	Mackenzie	I agree to the proposed new authorship shown in section 4		Institute of Medical Sciences, University of Aberdeen, Foresterhill, Aberdeen, AB25 2ZD, UK.	02/12/21

SPRINGER NATURE Springer Nature is one of the world's leading global research, educational and professional publishers, created in May 2015 through the combination of Nature Publishing Group, Palgrave Macmillan, Macmillan Education and Springer Science+Business Media.

Change of authorship request form (pre-acceptance)

7th author	Eileen	Bresnan	I agree to the proposed new authorship shown in section 4	Dr Eileen Bresnan	Marine Scotland Science, Marine Laboratory, 375 Victoria Road, Aberdeen, AB11 9DB, UK.	02/12/21
8th author	María	Segovia	I agree to the proposed new authorship shown in section 4	[illegible]	Department of Ecology, Faculty of Sciences, University of Málaga, Boulevard Louis Pasteur s/n, 29071-Málaga, Spain.	02/12/2021

Please use an additional sheet if there are more than 7 authors.

SPRINGER NATURE Springer Nature is one of the world's leading global research, educational and professional publishers, created in May 2015 through the combination of Nature Publishing Group, Palgrave Macmillan, Macmillan Education and Springer Science+Business Media.

Change of authorship request form (pre-acceptance)

Important information. Please read.

- Please return this form, fully completed, to Springer Nature. We will consider the information you have provided to decide whether to approve the proposed change in authorship. We may choose to contact your institution for more information or undertake a further investigation, if appropriate, before making a final decision.

- By signing this declaration, all authors guarantee that the order of the authors are in accordance with their scientific contribution, if applicable as different conventions apply per discipline, and that only authors have been added who made a meaningful contribution to the work.

- Please note, we cannot investigate or mediate any authorship disputes. If you are unable to obtained agreement from all authors (including those who you wish to be removed) you must refer the matter to your institution(s) for investigation. Please inform us if you need to do this.

- If you are not able to return a fully completed form within **30 days** of the date that it was sent to the author requesting the change, we may have to withdraw your manuscript. We cannot publish manuscripts where authorship has not been agreed by all authors (including those who have been removed).

- Incomplete forms will be rejected.
